# Navigating complex peptide structures using macrocycle conformational maps†

**DOI:** 10.1039/d2cb00016d

**Published:** 2022-04-19

**Authors:** Timothy J. McTiernan, Diego B. Diaz, George J. Saunders, Fiona Sprang, Andrei K. Yudin

**Affiliations:** Davenport Research Laboratories, Department of Chemistry, University of Toronto Toronto ON M5S 3H6 Canada andrei.yudin@utoronto.ca

## Abstract

Identification of turn motifs that are stabilized by intramolecular hydrogen bonds can be useful in describing the conformation of peptide systems. However, this approach is somewhat insufficient for cyclic peptides because peptide regions that are not positioned within a hydrogen bond can be left with no description. Furthermore, non-regular secondary structures and other rarely-observed conformations can be left without detailed evaluation. Herein, we describe “higher-order” *ϕ*/*ψ* plots termed macrocycle conformational maps (MCMs) as a tool for evaluating and comparing the conformations of a series of structurally related macrocyclic peptides.

## Abstract 2

Identification of turn motifs that are stabilized by hydrogen bonds can be useful in describing the conformation of peptides. Herein, we describe “higher-order” *ϕ*/*ψ* plots termed macrocycle conformational maps (MCMs) as a tool to evaluate and compare the conformations of related macrocycles.

## Graphical abstract

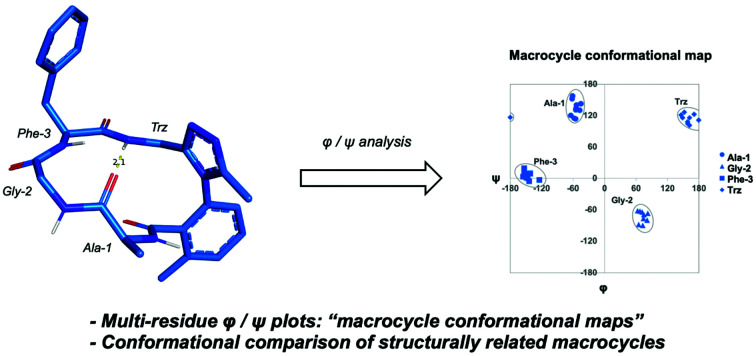

## Introduction

Macrocyclic peptides are prominent structures among natural products as well as synthetic bioactive molecules.^1–4^ Due to the number of rotatable bonds in their backbone, macrocyclic peptides can access a broad range of feasible conformations that are close in free energy.^5,6^ Relatively minor modifications to the structure of such systems can result in drastic conformational changes.^7–9^ The network of interacting structural elements in macrocyclic peptides gives rise to conformational organisation that is challenging to predict and characterise.^10–12^

**Fig. 1:**
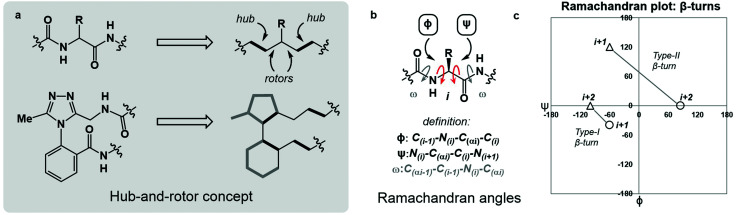
An overview of angular constraints in peptidic and non-peptidic backbones. (a) The hub-and-rotor concept; (b) definition of Ramachandran angles *ϕ*, *ψ* and *ω*; (c) plotting *ϕ* and *ψ* for *i* + 1 and *i* + 2 residues of both Type I and II β-turns illustrates their respective conformational maps.

In broad terms, macrocyclic peptide structures are distinguished for their “hub-and-rotor” architecture.^13–15^ In addition to fully rotatable bonds (rotors), the peptide backbone contains hub sub-structures that possess limited rotational opportunities. Backbone amide bonds generally adopt the *trans*-configuration (*ω* = 180°) and are considered hubs due to their restricted rotational freedom and planarity. Rotors in the backbone are described by the phi (*ϕ*) and psi (*ψ*) dihedral angles.^16–18^ In synthetically modified peptide systems, unnatural structural elements can also be judged based on their rotational properties (Fig. 1a). The global minimum energy conformation of a macrocyclic peptide structure arises from the dynamic interplay of hubs and rotors in the backbone, as these elements engage in coupled bond rotations.^7,19^ Coupling in bond rotations describes the phenomenon wherein the rotation of one bond in a molecule influences the rotation of neighbouring bonds, propagating conformational effects throughout the molecule as a function of the degree of rotational coupling.^20–22^

The Ramachandran plot is an analytical tool describing the geometry of the backbone rotors *ϕ* and *ψ* in proteins and peptides (Fig. 1b).^23^ The *ϕ*/*ψ* plot has been conventionally used for protein structure validation, and has recently found utility in the evaluation of both protein geometry and conformational trends among amino acids.^24–29^ Recently, Jayaram and coworkers demonstrated that the three-dimensional conformation of a protein or peptide can be reconstructed from an alphanumeric string representing both the *ϕ*/*ψ* coordinates and the structural identity of each amino acid in a sequence.^30^ In this context, “higher-order” *ϕ*/*ψ* plots representing sequential residues can be created to map the global conformation of peptide structures and to probe the local conformational effects of neighbouring residues. An illustrative example of such multi-residue *ϕ*/*ψ* plots can be found in the analysis of distinct β-turn types. Numerous β-turn types have been described. These motifs share the property of (*i* → *i* + 2) hydrogen bonding, but differ in the distribution of *ϕ*/*ψ* angles, which annotate the amino acid residues constrained within the turn and define the turn's type.^31^ Two-residue *ϕ*/*ψ* plots representing distinct β-turn types map the unique regions of conformational space occupied by each of these constructs (Fig. 1c).

We sought to apply the logic of *ϕ*/*ψ* conformational mapping to develop a system for classifying macrocyclic peptide structures. This approach became particularly meaningful after we outlined work on conformational “dark space” in macrocyclic peptides. As part of this investigation, we were able to stabilize certain conformations in solution that were difficult to describe in conventional terms. In general, dark space refers to metastable peptide conformations not frequently observed in nature. A specific example of dark space can be found when backbone amino acids exhibiting rare left-handed helical conformations are over-represented because of conformational restriction in a macrocycle.^32^ In order to observe dark space conformations on a relevant timescale, structural constraints must be integrated into macrocyclic systems. Accordingly, we developed a synthetic motif termed the dominant rotor. This motif bears an internal atropisomer exhibiting a large kinetic barrier to rotation (Δ*G*^‡^ > 27 kcal mol^−1^). Upon incorporation into a macrocycle, the dominant rotor gives rise to two isolable atropisomeric species that are different in free energy. The difference in free energy between atropisomers largely reflects the backbone's degree of strain resulting from the conformational restriction imparted by the dominant rotor. Overall, the dominant rotor allows isolation of metastable “dark space” macrocycles that can exhibit unconventional conformational behaviour.

Macrocyclic peptide systems can engage in intramolecular reactions that impact their conformation. The net conformation of a macrocyclic peptide represents an interplay of all stabilizing and destabilizing interactions in the system.^33^ The so-called composite barriers are associated with processes that induce coupled bond rotations. For instance, intramolecular chemical transformations in macrocycles such as the Boulton–Katritzky rearrangement can be used as tuneable barriers that impact the system's conformation.^13,34^ Atropisomerisation of dominant rotor-containing macrocycles can lead to another type of composite barrier when the high-energy rotation of the dominant rotor gets coupled to distant rotors in the system, giving rise to conformational differentiation.

The lack of robust methodology for describing macrocyclic peptide conformation is at the heart of the challenge in evaluating these constructs. Classically, “turn types” have dominated the discourse on macrocyclic peptide conformation. While classical turn types adequately describe some local regions of proteins and linear peptides, the cyclic nature of macrocyclic systems can render this approach inadequate. Exclusive evaluation of macrocyclic peptides according to their internal turn structures inevitably leaves regions of the ring that do not engage in classically defined turns without analysis. Furthermore, this approach ultimately leaves macrocycles that engage in non-regular secondary structures (NORS) without adequate options for conformational description.^35–37^ A comprehensive approach to evaluating macrocyclic peptide conformation must be established to meet these challenges.

In this paper, we define multi-residue graphical representations of macrocyclic peptide conformation as “macrocycle conformation maps” (MCMs). The sequential pattern of datapoints on the MCM describes the global conformation of a given macrocycle, and the coordinates of each individual datapoint describe the local conformation at each constituent amino acid. Furthermore, the conformational relationship between two macrocycles of identical ring size and related rotor structure can be described by their differences in MCM coordinates (ΔMCM). The difference in *ϕ*/*ψ* for each residue in a pair of macrocycles gives rise to a pattern of ΔMCM “vectors” corresponding to the dihedral angular differences between conformations. A vector oriented parallel to the *ϕ*-axis suggests that the conformational difference between species at this residue occurs primarily in the *ϕ*-dimension. Similarly, a vector oriented parallel to the *ψ*-axis represents a conformational difference occurring primarily in the *ψ*-dimension. The magnitude of a ΔMCM vector represents the magnitude of angular difference between analogous macrocycles but does not represent a mechanistic pathway of conformational interconversion in cases where such interconversion is possible. The use of MCMs and ΔMCMs allows for graphical evaluation of macrocycle conformation and identification of regions of conformational change among related macrocycles. Using MCM methodology we have evaluated a range of macrocyclic peptide conformations, including those that do not possess a hydrogen bond defining a classical turn structure.

## Results and discussion

### Synthesis of peptide macrocycles

We prepared a series of 16-membered dominant rotor-containing macrocyclic peptides. Each of these species bears the Ala-Gly-Phe (AGF) tripeptide motif constrained by a dominant rotor. Our scope sought to evaluate the divergent conformational properties of this motif upon integrating a single methyl group in a range of positions about the dominant rotor. In medicinal chemistry, the “magic methyl” effect describes the introduction of a methyl group that impacts the conformation of a drug candidate, consequently affecting binding affinity and/or selectivity for its ligand.^38^ Inspired by this logic, we introduced methyl groups in distinct positions and recorded the differences in global conformation induced by methylation. Macrocycles 1–4 were synthesized using solution phase DEPBT-mediated macrolactamisation of linear peptides obtained from solid-phase peptide synthesis (SPPS) (Table 1). The dominant rotor was introduced as a racemic mixture (1,2) or mixture of diastereomers (3,4). In principle, two macrocyclic atropdiastereomers can be formed for each system.

**Table 1:** Synthesis of dominant rotor-containing macrocycles. For full details, see the ESI

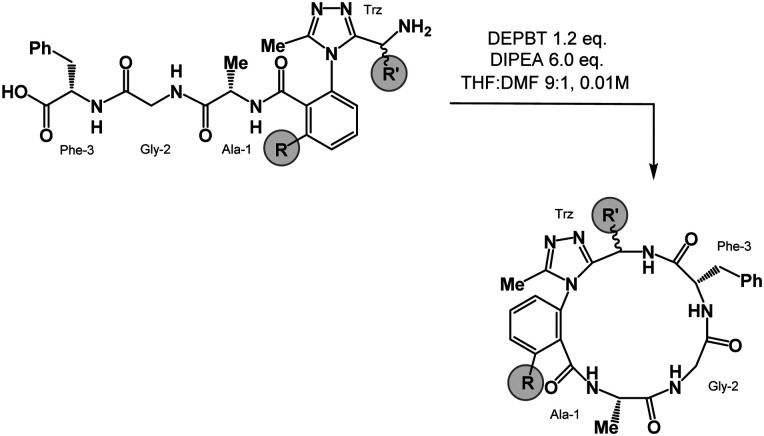				
Macrocycle	R	R′	*R*_a_ yield (%)	*S*_a_ yield (%)
1	H	H	26	24
2	Me	H	20	28
3	H	(*R*)-Me	—	31
4	H	(*S*)-Me	11^a^	—

a *R*_*a*_-4: Three minor species identified but not isolated after macrocyclisation.

### Understanding macrocyclic backbones using MCMs

Each of the macrocycles reported in Table 1 were fully characterized using ^1^H and ^13^C NMR spectroscopy. We performed variable temperature (VT) ^1^H NMR to calculate chemical shift/temperature coefficients for each amide NH in the molecule. A temperature coefficient of less than 4 ppb K^−1^ typically indicates that a proton is involved in hydrogen bonding or is not exposed to solvent.^39^ In addition, distance restraints were calculated using correlations in 2D ROESY spectra. These distances were used to generate structures that then underwent 100 ns molecular dynamics (MD) simulations in DMSO.^40,41^ From these MD simulations, cluster analysis was performed to provide the ten most populated clusters generated from the simulation, which were checked for distance restraint violations. The dihedral angles of each structure were measured and used for MCM analysis. Full experimental procedures, computational details and experimental data can be found in the ESI†.

**Fig. 2:**
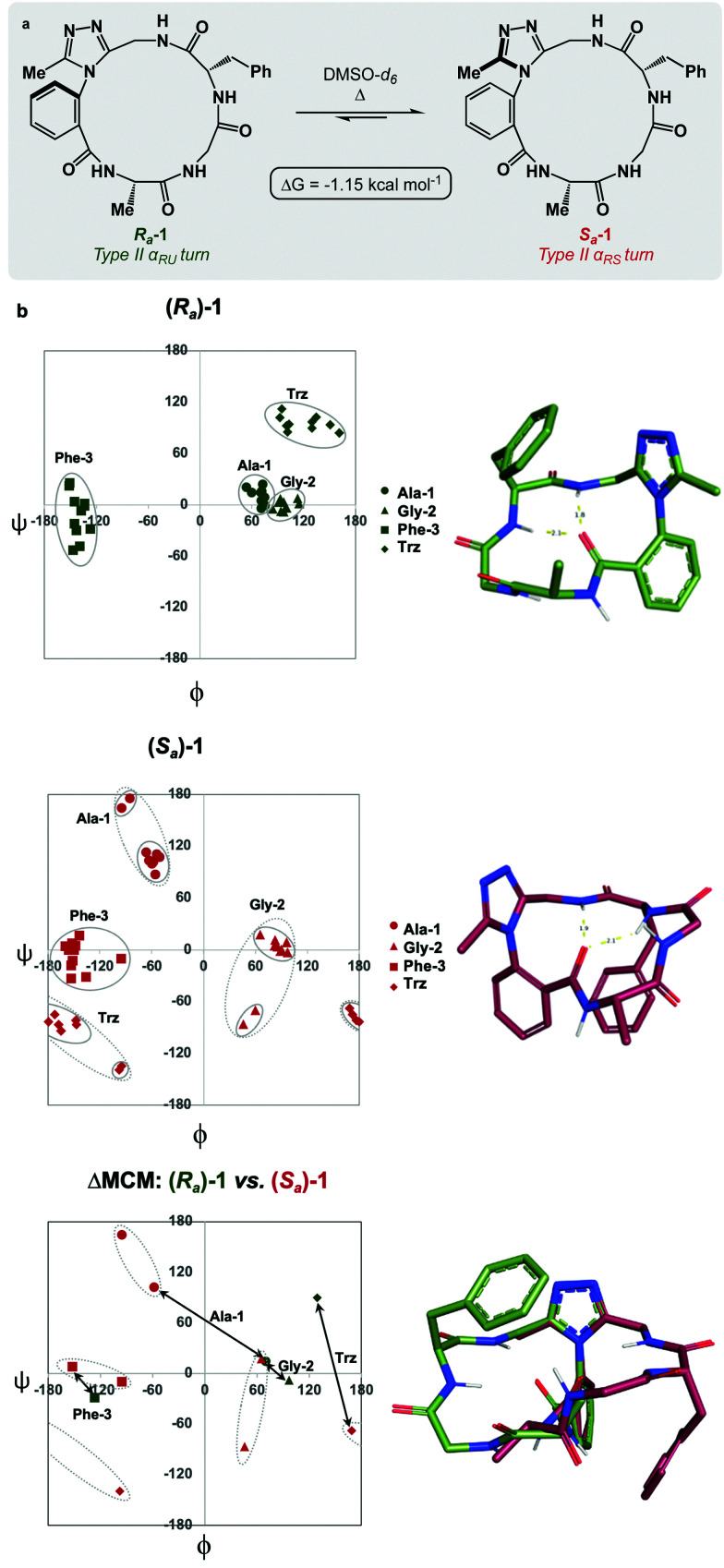
MCM analysis reveal conformational differences between *R*_*a*_-1 and *S*_*a*_-1. (a) 16-membered dominant rotor cyclic peptides *R*_*a*_- and *S*_*a*_-1; (b) MCMs and 3D representations of *R*_*a*_- and *S*_*a*_-1; ΔMCM representing conformational differences between *R*_*a*_-1 and *S*_*a*_-1. MCMs display single-residue conformational ensembles bounded by solid ovals; major and minor clusters of *S*_*a*_-1 are jointly enclosed by dashed ovals.

Macrocycle 1 was synthesized as a mixture of atropdiastereomers, *R*_*a*_-1 and *S*_*a*_-1, which were individually isolated. To understand the differences in free energy between the two structures, the equilibrium constant for the interconversion between the two atropdiastereomers was calculated by performing a thermal equilibration experiment followed by subsequent analysis using VT-NMR. The entropic and enthalpic components were extrapolated using a Van’t Hoff plot (see the ESI†).^32^ This analysis established that *S*_*a*_-1 was lower in free energy than *R*_*a*_-1 by 1.15 kcal mol^−1^ (Fig. 2a). Classical turn structure evaluation concluded that the dark-space *R*_*a*_-1 contained two nested intramolecular hydrogen bonds defining a Type II α_RU_ turn. Similarly, the major conformer of *S*_*a*_-1 contained two intramolecular hydrogen bonds defining a Type II α_RS_ turn. In a study on the classification of 356 α-turns observed in natural proteins, only 28 were found to be Type II α_RU_ turns, representing approximately 8% of the structures in this dataset. Furthermore, 39 α-turns were found to be Type II α_RS_ turns, representing approximately 11% of these structures.^42^

MCM analysis was undertaken for both *R*_*a*_-1 and S_*a*_-1 to understand specific structural differences between the two macrocycles. The dihedral angles derived from the ten most populated clusters after 100 ns MD simulations were plotted (Fig. 1b). MCM analysis of *R*_*a*_-1 revealed that the ring adopts a single conformation with relatively tight *ϕ*/*ψ* distribution over the course of the MD simulation. Both of Ala-1 and Gly-2 occupy the positive *ϕ*-space in *R*_*a*_-1. The positioning of Ala-1 in positive *ϕ*-space represents a non-ground-state conformation, as this sidechain incurs 1,3-allylic strain with the neighbouring amide. Evaluation of the Ramachandran plot of *S*_*a*_-1 revealed that the ring features more conformational mobility over the course of the MD simulation, adopting two related conformations. Furthermore, Gly-2 is the only residue to occupy positive *ϕ*-space in this structure. The positioning of all l-amino acids in low-energy negative *ϕ*-space in *S*_*a*_-1 compared to *R*_*a*_-1 reflects Δ*G* between these species.

*R*_*a*_-1 and *S*_*a*_-1 are atropdiastereomers and can thus be conformationally compared against one another. ΔMCM plots overlay the lowest-violation cluster from each MD simulation on the same plot and establish a dihedral angular comparison of analogous macrocycles when ring size and amino acid substituents are held constant. In cases where multiple conformations were identified over the course of an MD simulation, dashed ovals joined the multiple species, and a vector was drawn only to the most populated species. ΔMCM vectors were drawn between the corresponding residues to represent the conformational changes undergone by each residue of *R*_*a*_-1 and *S*_*a*_-1 (Fig. 2b, bottom). The angular changes undergone by both Gly-2 and Phe-3 are relatively minor and are reflected in the vectors of lesser magnitude representing both residues. In comparison, the Ala-1 vector is significant in magnitude, traversing the *ϕ* = 0 axis and representing the localisation of much of the conformational change between the atropisomers. This vector indicates that between *R*_*a*_-1 and *S*_*a*_-1, the change in AGF conformation is largely attributable to the *ϕ*-axis of Ala-1. The dihedral angular change characterized by the Ala-1 vector naturally reflects the change in α-turn type from Type II α_RU_ in *R*_*a*_-1 to Type II α_RS_ in *S*_*a*_-1. MCMs and ΔMCMs provide a view of the peptide's conformation that more fully encompasses both its angular structure, conformational plasticity, and relationship to analogues over the course of their respective MD simulations.

**Fig. 3:**
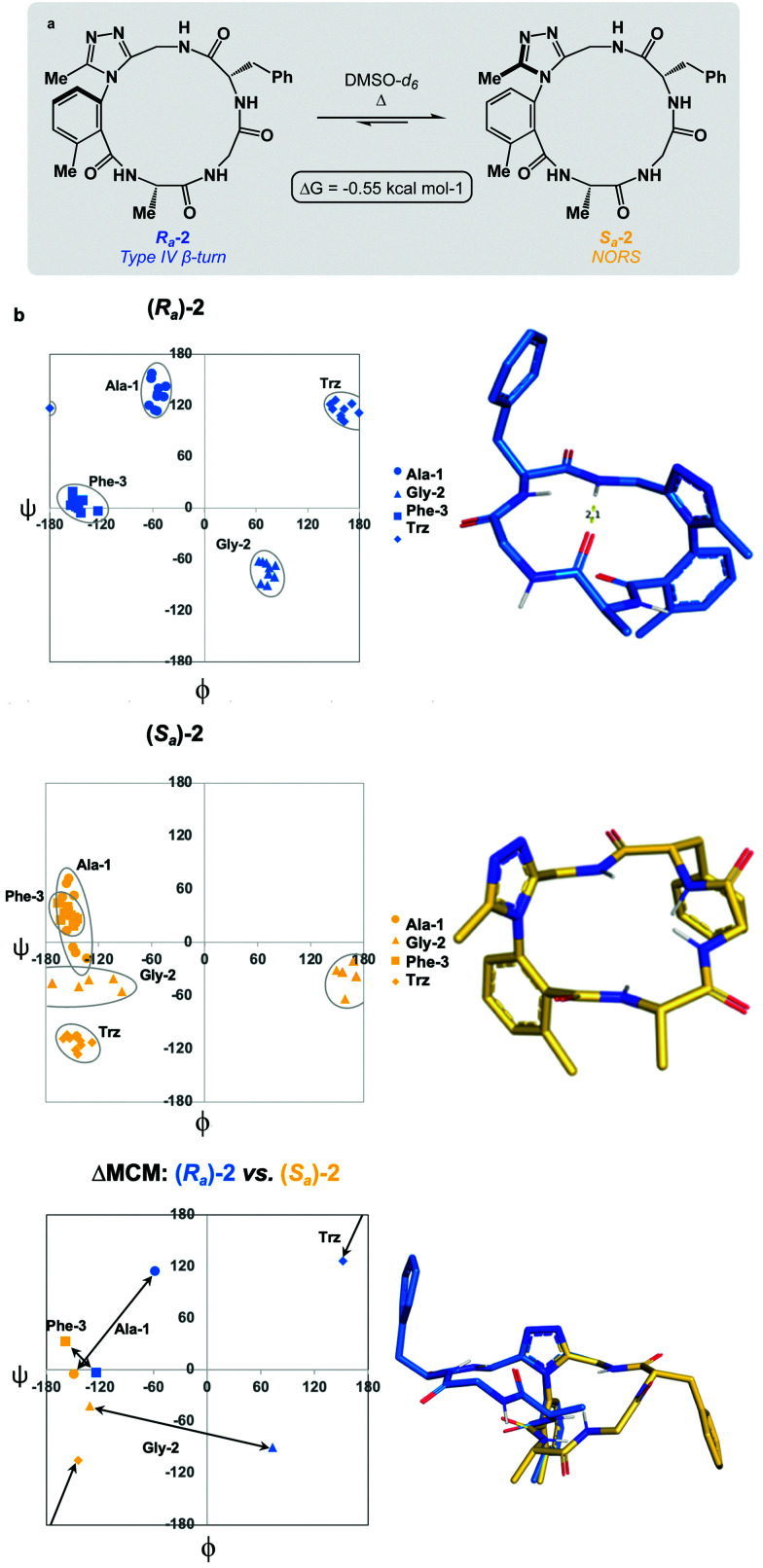
MCM analysis reveals conformational flexibility of Gly-2 in *S*_*a*_-2. (a) *R*_*a*_-2 and *S*_*a*_-2; (b) MCMs and 3D representations of *R*_*a*_- and *S*_*a*_-2; ΔMCM representing conformational differences between *R*_*a*_-2 and *S*_*a*_-2. MCMs display single-residue conformational ensembles bounded by solid ovals.

We sought to interrogate the effects of substitution to the dominant rotor through a “methyl scan”, wherein the location and/or chirality of methyl substituents on the dominant rotor are varied. We hypothesised that this approach might allow us to observe the ensemble of conformations available to an oligopeptide constrained in a macrocycle, and to finely tune the conformation based on this information. Toward this goal, we designed macrocyclic peptides 2–4, varying the location and/or chirality of methyl groups on the dominant rotor. To understand the downstream effects of methyl placement on the dominant rotor, we prepared macrocycle 2 bearing a methyl group on the *meta* position of the aryl ring. The atropdiastereomers *R*_*a*_-2 and *S*_*a*_-2 were identified and individually isolated. VT NMR experiments to calculate *K*_eq_ and Δ*G* for the equilibrium between the two species established that *S*_*a*_-2 was lower in free energy than *R*_*a*_-2 by 0.55 kcal mol^−1^ (Fig. 3a). Using classical turn evaluation, we concluded that the “dark-space” *R*_*a*_-2 adopts a Type IV β-turn. This type of β-turn corresponds to a backbone conformation that is not conventionally defined.^43^ Interestingly, *S*_*a*_-2 is not possible to classify using conventional terminology because it does not contain an intramolecular hydrogen bond and is thus designated as a non-regular secondary structure (NORS). As the individual AGF conformation of both *R*_*a*_-2 and *S*_*a*_-2 cannot be defined by classical terminology, these “dark-space” conformations require description through their relationship of sequential dihedral angles.

Curiously, the MCM of *S*_*a*_-2 illustrates the dynamic coupling of rotors through the distribution of adjacent *ϕ*/*ψ* coordinates of Ala-1 and Gly-2 (Fig. 3b). Over the course of the MD simulation, this region of the ring was found to be conformationally mobile, with the distribution of *ϕ*/*ψ* coordinates extending along the *ψ*-axis for Ala-1 and along the *ϕ*-axis for Gly-2. This finding represents the high conformational mobility of the Ala-1 amide plane over the course of the MD simulation. It also demonstrates the fine detail in conformational dynamics that can be elucidated using MCMs. The use of MCMs not only permits classification of the unconventional conformation of *S*_*a*_-2, but also illustrates its regions of high conformational mobility. ΔMCM screening reveals that the angular differences in AGF conformation between *R*_*a*_-2 and *S*_*a*_-2 are primarily localized at Ala-1 and Gly-2. Ala-1 occupies distinct coordinates in the negative *ϕ*-space in both structures and Gly-2 undergoes a large angular change, crossing the *ϕ* axis. Notably, both conformations are distinct from those of *R*_*a*_-1 and *S*_*a*_-1 despite their structural similarity.

As the dominant rotor's *meta*-methyl constitutes the only structural difference between 1 and 2, the conformational differences observed can be attributed to its downstream effects. In particular, the methyl group perturbs the ring's rotor structure by clashing with the proximal benzamide motif. This perturbation changes the benzamide rotor's orientation, resulting in downstream conformational effects due to coupling in bond rotations. In general, the most stable dihedral angle for a benzamide motif is *θ* = ±30/150°.^44^ Macrocycle 1 adopts dihedral angles that align with the most stable ones: *R*_*a*_-1*θ* = −141°; *S*_*a*_-1*θ* = 143°. As a result of structural perturbation by the rotor methyl, *R*_*a*_-2*θ* = −117°; *S*_*a*_-2*θ* = −76°. The global conformational differences in 2 in relation to 1 stem from the downstream effects of this benzamide bond rotation.

**Fig. 4:**
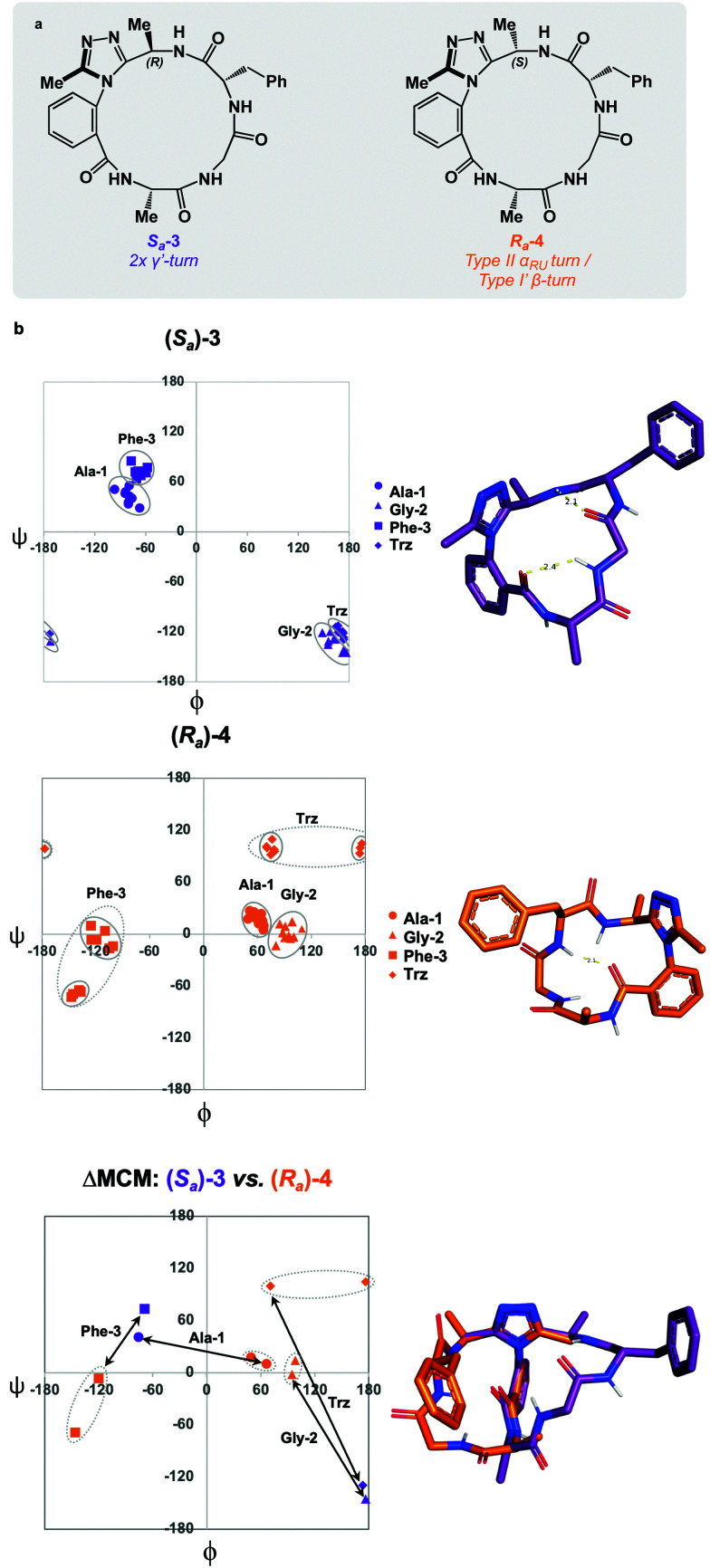
Comparison of *S*_*a*_-3 and *R*_*a*_-4 using MCM methodology. (a) *S*_*a*_-3 and *R*_*a*_-4; (b) MCMs and 3D representation of *S*_*a*_-3 and *R*_*a*_-4; ΔMCM representing conformational differences between *S*_*a*_-3 and *R*_*a*_-4. MCMs display single-residue conformational ensembles bounded by solid ovals. Major and minor clusters of *R*_*a*_-4 are jointly enclosed by dashed ovals.

To complete the methyl scan of the dominant rotor, both (*R*)- and (*S*)-methyl groups were individually introduced in the vicinity of the triazole fragment (Fig. 4a). We hypothesized that a chiral sidechain in this position may sterically clash with the dominant rotor in one well, but not the other. In this way, the *R*_*a*_ : *S*_*a*_ product distribution could be controlled through the introduction of a chiral centre on the dominant rotor, wherein the chirality may influence the distribution of atropisomers. We first tested this theory by incorporating a (*R*)-methyl group on the dominant rotor to form a 16-membered AGF macrocycle. Excitingly, a single species was observed upon macrocyclization, and *S*_*a*_-3 was isolated as a single species (Fig. 4a). Conformational evaluation of *S*_*a*_-3 revealed that it formed a largely rigid conformation bearing two intramolecular hydrogen bonds, classically defining γ′-turns about each of Ala-1 and Phe-3 (Fig. 4b). In a similar experiment, the point chirality at this position was changed to (*S*), yielding the 16-membered AGF macrocycle 4. One major species was isolated and assigned *R*_*a*_-4 (Fig. 4a). Alongside the major species, three minor species were identified by LC-MS analysis but were not analyzed in detail. Conformational evaluation of *R*_*a*_-4 revealed a set of two related conformations, one bearing a Type II α_RU_ turn and the other bearing a Type I′ β-turn. This effect was intriguing, as the introduction of the (*S*)-methyl group in *R*_*a*_-4 resulted in a conformational deviation from the methylene-bearing *R*_*a*_-1.

ΔMCM screening of *S*_*a*_-3 and *R*_*a*_-4 reveals conformational differences at each residue. Ala-1 traverses the *ϕ* = 0 axis between the two species. Gly-2 occupies positive *ϕ*-space in both species, yet occupies distinct regions in each. Furthermore, Phe-3 occupies two unique regions of the negative *ϕ*-space in each conformer. This plot illustrates the potential value in the introduction and screening of chiral substituents on the dominant rotor. We found that the point chirality at this position can bias the atropisomer formed, resulting in divergent conformations of the same peptide sequence. This further displays the conformational tuning and downstream effects that can be achieved by modifying the dominant rotor itself.

**Fig. 5:**
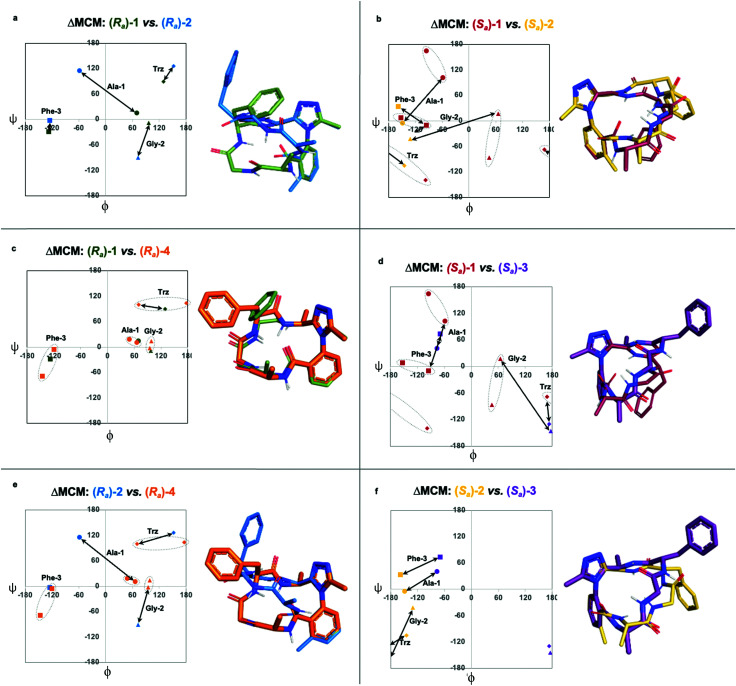
Further comparison of pairs of dominant rotor containing macrocycles using ΔMCM analysis. ΔMCMs: (a) *R*_*a*_-1*vs.R*_*a*_-2; (b) *S*_*a*_-1*vs.S*_*a*_-2; (c) *R*_*a*_-1*vs.R*_*a*_-4; (d) *S*_*a*_-1*vs.S*_*a*_-3; (e) *R*_*a*_-2*vs.R*_*a*_-4; (f) *S*_*a*_-2*vs.S*_*a*_-3. Dashed ovals represent major and minor conformations of *S*_*a*_-1. Points on the ΔMCMs represent the most populated cluster of each species. Arrows represent dihedral angular changes.

We undertook ΔMCM analysis for each of the macrocycles in the methyl scan to highlight conformational differences between these analogues. Since *R*_*a*_-1 and *R*_*a*_-2 share the same ring size and similar hub/rotor structure, these species can be appropriately evaluated using this method. An overlay of *ϕ*/*ψ* coordinates for both rings reveal a range of conformational differences that arise downstream from the methyl group's perturbation of the benzamide rotor (Fig. 5a). The most dramatic difference between these two species is that Ala-1 occupies a positive *ϕ*-angle in *R*_*a*_-1 but a negative *ϕ*-angle in *R*_*a*_-2. The methyl group perturbs the benzamide dihedral angle to turn into the ring, allowing the peptide chain to relax at Ala-1.

A similar assessment can be made to evaluate the conformational differences between *S*_*a*_-1 and *S*_*a*_-2 (Fig. 5b). In this example, the dihedral angular differences are again localized primarily at Ala-1 and Gly-2. This conformational effect is similar to that in the *R*_*a*_-well, as the dominant rotor's aryl methyl group perturbs the benzamide rotor. The bond rotation induces downstream conformational effects that propagate throughout the system but are primarily localized to those residues most proximal to the dominant rotor.

ΔMCM screening of *R*_*a*_-1 and *R*_*a*_-4 reveals that the conformations of these systems are rather similar (Fig. 5c). This is not surprising, as these systems bear related rotor structure. A curious component of the behaviour of *R*_*a*_-4 is its conformational dynamics. *R*_*a*_-4 can access both a Type II α_RU_ turn and a Type I′ β-turn. These conformations are similar, differing primarily in the identity of the hydrogen bonding NH: hydrogen bonding of Trz-NH leads to a Type II α_RU_ turn and hydrogen bonding of Phe-3-NH leads to a Type I′ β-turn. These conformations are under exchange in the MD simulation, reflecting the component of conformational instability introduced by the (*S*)-methyl of the dominant rotor of *R*_*a*_-4 in comparison to the methylene of *R*_*a*_-1. This effect illustrates that relative conformational dynamics can also be evaluated using the ΔMCM plot.

ΔMCM screening allows the comparison of *S*_*a*_-1 with *S*_*a*_-3 (Fig. 5d). These structures differ only in the placement (*R*)-methyl in *S*_*a*_-3. This minor structural change gives rise to significant conformational differences. Angular changes are observed in each residue, wherein both Ala-1 and Phe-3 adopt *ϕ*/*ψ* regions characteristic of γ′-turns with Gly-2 occupying an extended conformation in *S*_*a*_-3. This is compared to the Type II α_RS_ turn adopted by the major conformer of *S*_*a*_-1. The comparison of *S*_*a*_-2 with *S*_*a*_-3 constitutes a particularly interesting example, as these species have the same dominant rotor geometry but differ in the position of the methyl group (Fig. 5f). While each species adopts a conformation characterized by all-negative *ϕ*-angles, each conformation presents as stable over the course of the MD simulation and each residue occupies distinct regions that characterize their respective turn structure.

### Analysis of non-dominant rotor macrocycles

**Fig. 6:**
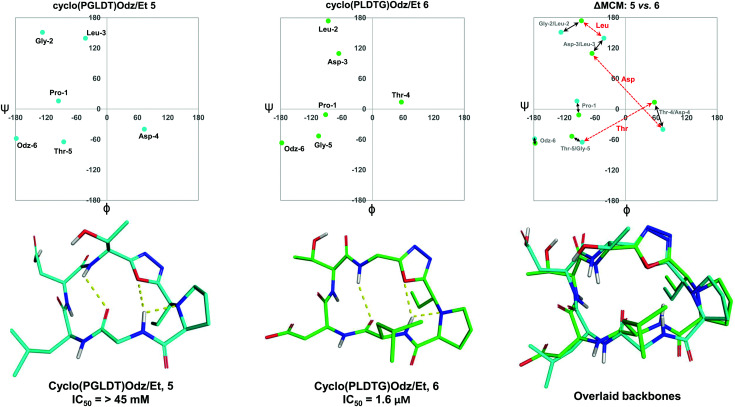
MCM analysis of the solution structures of cyclo(PGLDT)Odz/Et 5 and cyclo(PLDTG)Odz/Et 6 reveal conformational differences that pertain to biological activity. Points on the maps represent the *ϕ*/*ψ* coordinates of the most populated cluster of each species. Black arrows represent dihedral angular changes between residues in same relative position around the macrocycle *e.g.*, Gly-2/Leu-2. Red dashed arrows represent dihedral angular changes between the same residue in different positions around the macrocycle, *e.g.*, Thr-5/Thr-4.

To further demonstrate the utility of MCM plots, we performed analysis on other macrocyclic structures possessing both a disparate ring size and distinct hub and rotor profile. Specifically, we wanted to see if MCM methodology could be used to understand conformational nuances in biologically relevant molecules, with the downstream possibility to aid in the design of related bioactive molecules. The interaction between the lymphocyte integrin α_4_β_7_ and the mucosal address in cell adhesion molecule-1 (MAdCAM-1) have been implicated in several diseases such as Type 1 diabetes, Crohn's disease and chronic inflammatory bowel disease.^45–47^ The binding of the integrin to MAdCAM-1 relies on the Leu-Asp-Thr (LDT) tripeptide sequence located on the *N*-terminal Ig domain of MAdCAM-1, which has stimulated the development of inhibitors based on this motif.^48,49^ Previously reported macrocycles 5 and 6 each contain the LDT sequence in a unique backbone position, and incorporate a 1,3,4-oxadiazole (Odz).^40^ The solution structures of 5 and 6 reported in DMSO (derived from 10 ns MD simulations) each include a Type II β-turn (Fig. 6). Furthermore, the reduced amide and oxadiazole motif stabilizes a β-turn-like conformation with the proximal amide NH. Despite their similar backbone conformations, positional isomers 5 and 6 display significantly different inhibitory activity towards the MAdCAM-1/α_4_β_7_ protein-protein interaction (measured by ELISA assays: 5, IC_50_ = >45 mM; 6, 1.6 μM).^40^

To visualise the specific differences between the two molecules, individual MCM plots of 5 and 6, as well as a ΔMCM plot comparing the two structures were prepared from previously reported dihedral angles extracted from the most populated cluster of 10 ns MD simulations (Fig. 6).^40^ Here, we show two sets of vectors in the ΔMCM plot: (i) black solid arrows represent the changes in dihedral angle between specific positions around the macrocycle, *e.g.* Gly-2/Leu-2, and (ii) red dashed arrows represent dihedral angular changes between the specific Leu/Asp/Thr residues as a result of movement within the macrocyclic backbone, *e.g.* Thr-5/Thr-4.

The ΔMCM plot comparing 5 and 6 presents solid black vectors of relatively small magnitude for each residue in the backbone, which highlights the conformational similarities between the two macrocycles. The red dotted vectors displaying movement of the LDT residues within the backbone show that the *ϕ*/*ψ* space occupied by the Leu residue does not change dramatically based on its relative position in the turn sequence. In contrast, the Asp and Thr residues occupy distinct regions of conformational space in these two molecules. While it is challenging to identify generalisable conformational characteristics that distinguish high-affinity inhibitors from a single example, we believe that within the context of a broader dataset this type of analysis may prove fruitful in highlighting conformational regions of interest for the design of macrocyclic peptide-based scaffolds. This analysis demonstrates the use of our methodology to visualise conformational relationships in macrocycles with bioactivity, which we anticipate will be of immediate utility to chemists developing other macrocyclic peptide inhibitors.

The findings from these ΔMCMs illustrate the breadth of conformations available to short oligopeptide sequences constrained in macrocyclic scaffolds. A current challenge in macrocycle development entails the use and discovery of specific modifications which enable rational and predictable access to specific areas of conformational space. Although this goal has not yet been achieved, we anticipate that future use of the MCM tool will enable better understanding of the conformational nuances caused by synthetic modifications to macrocyclic peptide backbones.

## Conclusions

MCM and ΔMCM screening can be useful tools to evaluate conformational ensembles identified for macrocyclic peptides and their structural analogues. During our study, we found that a “methyl scan” on dominant rotor fragments in macrocycles can result in significant differences in conformation that are adequately expressed using our method. Additionally, we established that by controlling the chirality of dominant rotor methylation, we can bias the system to favour a specific atropisomer. Furthermore, the dark-space conformations identified in this study were evaluated with MCM analysis, including those with non-regular secondary structures. In this case, MCMs allow for analysis of macrocyclic peptide structures that could not be defined using conventional turn-structure terminology.

ΔMCM screening can be a useful tool for the comparison of macrocyclic peptide conformations and should find utility in conformation-activity studies. This manner of analysing macrocyclic peptides could find applications in tuning the conformation of a series of structurally related systems. Such an approach could result in the design of more potent and selective macrocycle-based medicines as the functional properties of such compounds are intrinsically related to their conformation.

## Author contributions

A. K. Y. and T. J. M. conceived the study and designed the experiments; T. J. M., D. B. D., G. J. S., and F. S. carried out macrocycle synthesis, purification and characterisation, T. J. M. and D. B. D. computed the NMR-based structures and performed unrestrained MD simulations, T. J. M. and G. J. S. wrote the paper with help from all the authors.

## Conflicts of interest

There are no conflicts of interest to declare.

## Supplementary Material

CB-003-D2CB00016D-s001
