# Supplementary material for: Navigating complex peptide structures using macrocycle conformational maps
Source: RSC Chem Biol. 2022 Apr 19;3(6):739–47. doi: 10.1039/d2cb00016d (PMC9175111; doi:10.1039/d2cb00016d)
Supplement: CB-003-D2CB00016D-s001 [file CB-003-D2CB00016D-s001.pdf]

Electronic Supplementary Material (ESI) for RSC Chemical Biology.
This journal is © The Royal Society of Chemistry 2022

Navigating complex peptide structures using macrocycle conformation maps

Timothy J. McTiernan,[1] Diego B. Diaz,[1] George J. Saunders,[1] Fiona Sprang,[1] Andrei K. Yudin[1*]

[1] Department of Chemistry, University of Toronto, 80 St. George Street, Toronto, ON, M5S 3H6, Canada.

*Correspondence to: andrei.yudin@utoronto.ca

Table of Contents

1 Experimental 2

1.1 General experimental considerations 2

1.2 Synthesis of Dominant Rotors and Macrocycles 4

1.3 Previously reported compounds 7

1.4 Compound characterisation 7

1.5 Variable-Temperature NMR 12

1.6 Structure determination 18

1.7 NOE-derived distance restraints and calculated distances 20

1.8 Measurement of ϕ / ψ torsion angles for macrocycles 22

1.9 Van't Hoff analysis of dominant rotor macrocycles 25

2 Appendix A: NMR Spectra 28

2.1 NMR Spectra of Dominant Rotors 28

2.2 NMR Spectra of Macrocyclic Peptides 32

2.3 References 39

1 Experimental

1.1 General experimental considerations

General Information: Methylene chloride (DCM), methanol (MeOH) and acetonitrile (MeCN) were distilled from CaH_2 under nitrogen. Toluene was purified *via* solvent purification system. Tetrahydrofuran (THF) was freshly distilled from sodium benzophenone ketyl. All other solvents were of reagent grade quality and dried over 4Å MS prior to use. All reagents were purchased from commercial sources and used as received. Linear peptide precursors were synthesized by Fmoc solid-phase based peptide synthesis using 2-chlorotrityl chloride resin and double coupling steps with HATU. Amino acid reagents were sourced from AAPPTec LLC, Louisville, Kentucky, USA and P3 BioSystems, LLC, Shelbyville, Kentucky, USA. Peptide grade DIPEA was sourced from Sigma Aldrich (Oakville, ON). Reagent grade NMP and DMF were sourced from Caledon Laboratories Ltd., Georgetown, Ontario, Canada.

Chromatography: Flash column chromatography was carried out using Silicycle 230-400 mesh silica gel, or ISCO Teledyne Combiflash R_f 200 Flash system. Thin-layer chromatography (TLC) was performed on Macherey Nagel pre-coated glass backed TLC plates (SIL G/UV254, 0.25 mm) and visualized using a UV lamp (254 nm) or $KMnO_4$ dip. Reverse-phase chromatography was carried out using Redi*Sep* Rf Gold C_{18} Columns.

Nuclear Magnetic Resonance Spectroscopy: ^{1}H NMR, ^{13}C, and 2D-NMR spectra were recorded on Varian/Bruker 400 MHz, 500 MHz, 600 MHz or 700 MHz spectrometers. Samples of macrocycles for NMR spectroscopic studies were prepared by dissolving 4-5 mg of the compounds in 200 μL of DMSO-*d6* in a 3 mm borosilicate NMR tube. ^{1}H, ^{13}C, gCOSY, zTOCSY, ROESY,

$^{1}H,^{13}C$-gHSQC, and $^{1}H,^{13}C$-HMBC or $^{1}H,^{13}C$-CIGARAD NMR experiments were recorded and used to confirm the cyclic topology of each molecule. ^{1}H NMR spectra were referenced to DMSO-*d6* (δ 2.50 ppm). ^{13}C NMR spectra were referenced to DMSO-*d6* (δ 39.52 ppm). Mixing times for zTOCSY spectra were 80 ms, for ROESY spectra 150 ms. gHSQC spectra were recorded with a direct proton carbon coupling constant of 140 Hz, and gHMBC spectra with a long-range $^{1}H^{13}C$ coupling constant of 7 Hz. 2D Spectra were apodized using exponential and square sine window functions. Spectral data is reported as follows: chemical shift, multiplicity (s = singlet, d = doublet, t = triplet, q = quartet, dd = doublet of doublets, dt = doublet of triplets, ddt = doublet of doublet of triplets, dtd = doublet of triplet of doublets, m = multiplet, br = broad), coupling constant (*J*) in Hertz (Hz), and integration.

Mass Spectroscopy: High resolution mass spectra were obtained on a VG 70- 250S (double focusing) mass spectrometer at 70 eV or on an ABI/Sciex Qstar mass spectrometer with ESI source, MS/MS and accurate mass capabilities.

RP-HPLC/MS: Low-resolution mass spectra (ESI) were collected on an Agilent Technologies 1200 series HPLC paired to a 6130 Mass Spectrometer. Compounds were resolved on Phenomenex's Kinetex 2.6u C18 50x4.6mm column at room temperature with a flow of 1 mL/min. The gradient consisted of eluents A (0.1% formic acid in double distilled water) and B (0.1% formic acid in HPLC-grade acetonitrile). *Method A*: A linear gradient starting from 5% of B to 95% over 4 min at a flow rate of 1.0 mL/min. Stays constant at 95% for 1 min and then returns to 5% over 0.5 min. *Method B*: Stays constant at 5% of B for 0 min at a flow rate of 1.5 mL/min, followed by a linear gradient to 95% over 4.0 min. Stays constant at 95% of B for 3.0 min and then returns to 5% B over 0.5 min. *Method C*: A linear gradient starting from 5% of B to 95% over

15 min at a flow rate of 1.0 mL/min. Stays constant at 95% for 1 min and then returns to 5% over 0.5 min.

1.2 Synthesis of Dominant Rotors and Macrocycles

The synthesis of Fmoc-protected dominant rotor building blocks was accomplished through condensation of a Fmoc-amino acid hydrazide and a 1,3-benzoxazine.

Fmoc-amino acid hydrazide synthesis: Under a nitrogen atmosphere, the desired Fmoc-amino acid (1.0 eq) was stirred in dry THF (0.1 M) and the mixture was cooled to -15 °C in an ice/NaCl bath. *N*-methyl morpholine (1.5 eq) and isobutyl chloroformate (1.0 eq) were added to solution, and the mixture was stirred for 5 minutes. Boc-hydrazide (1.1 eq) was added dropwise as a solution in dry THF. The mixture was allowed to stir for 2 hours and warm to rt. The precipitate was then filtered off and the solvent was removed by rotary evaporation. The resultant Boc-protected Fmoc-amino acid hydrazide was then purified by flash chromatography (1:1 EtOAc:hexanes). The resultant material was treated with 1:1 *v/v* TFA:DCM and stirred for 1 hour. The mixture was concentrated *in vacuo* and the residue was extracted from $NaHCO_3/CHCl_3$ three times. The Fmoc-amino acid hydrazide is generally sparingly soluble in $CHCl_3$. The organic layers were mixed, dried over magnesium sulfate and the solvent was removed *in vacuo*.

1,3-benzoxazine synthesis: Anthranilic acid or a structural analogue was dissolved in excess acetic anhydride and the mixture was refluxed for 8 hours. The acetic anhydride was removed *in vacuo* to yield a dry solid. The resultant material washed with dry hexanes and filtered. Solids were collected, dried under high vacuum, and stored under argon. This class of molecule is water-sensitive and must be stored under inert conditions.

Dominant rotor synthesis: The dominant rotor was prepared by a condensation of Fmoc-amino acid hydrazide and 1,3-benzoxazine. To a flame-dried round bottom flask was added 1,3-benzoxazine (1.0 eq), dry MeCN, and activated 3 Å molecular sieves. Fmoc-amino acid hydrazide (1.0 eq) was added dropwise as a solution in dry MeCN. The mixture was stirred at 80 °C for 12 h. The mixture was then cooled to room temperature and dried *in vacuo*. The crude residue was purified by reverse phased C18 column chromatography with a gradient from 5:95 MeCN:H_2O to 95:5 MeCN:H_2O. The fractions containing the product were combined and lyophilized to give the heteroaryl-aryl acid as a white or off-white powder.

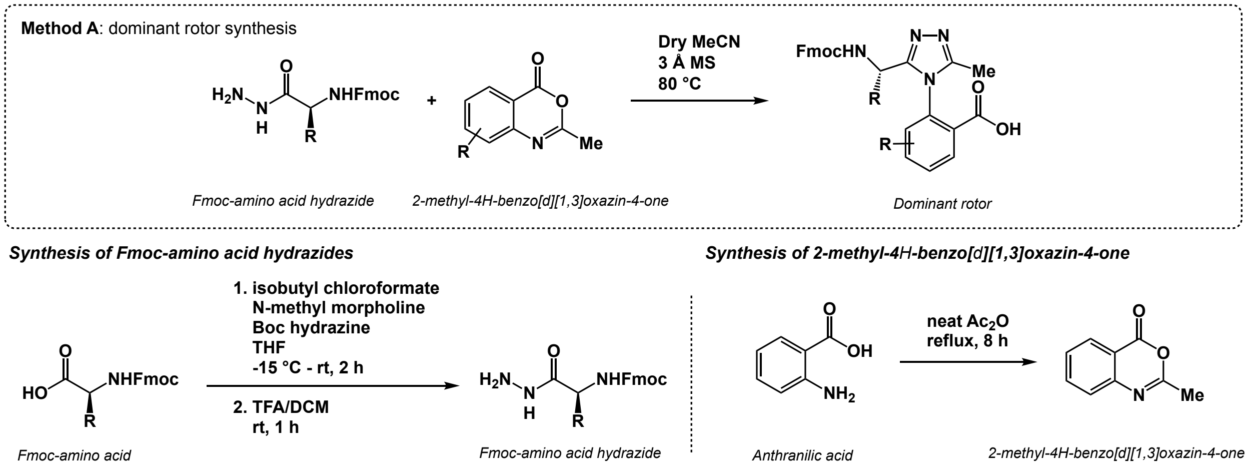

General procedure for the synthesis of dominant rotor macrocycles: Linear peptides containing the dominant rotor building block were assembled using Fmoc SPPS on 2-chlorotrityl resin. **Preparation of resin:** Fmoc-amino acid (1.2 eq. with respect to resin loading capacity) was dissolved in CH_2Cl_2 (10 mL/g resin). DIPEA was added (2 eq. with respect to the amino acid) and the solution was sonicated until fully dissolved. The 2-chlorotrityl resin was allowed to swell in CH_2Cl_2 (5 mL/g of resin) for 15 min. The CH_2Cl_2 was then drained. The Fmoc amino acid solution was added to the vessel containing the 2-Cl Trt resin and the vessel was agitated for 5 min. Another 4 eq. of DIPEA was then added and the vessel was left to agitate for an additional 60 min. The

resin was then treated with methanol (1 mL/g of resin) to endcap any remaining reactive 2-Cl Trt groups. The solution was mixed for 15 min, drained, and then rinsed with CH_2Cl_2 (x3) and DMF (x3). The resin was then used towards the preparation of a linear peptide. **Preparation of linear peptide sequence via manual synthesis:** Fully protected resin-bound peptides were synthesized via standard Fmoc solid-phase peptide chemistry manually. All *N*-Fmoc amino acids were employed. **Fmoc deprotection:** The resin was treated with 20% piperidine in DMF *v/v* twice, for 10 and 20 min respectively, with consecutive DMF and CH_2Cl_2 washes after each addition. **Fmoc amino acid coupling:** The resin was treated with 3 eq. of Fmoc amino acid, 3 eq. of HATU, and 6 eq. of DIPEA in DMF for 60 min. For difficult couplings, a second treatment with 3 eq. of Fmoc amino acid, 3 eq. of HATU, and 6 eq. of DIPEA in DMF for 40 min was employed. For unnatural Fmoc amino acids including dominant rotors, the resin was treated with 1.2 eq. of the respective Fmoc amino acid, 1.2 eq. of HATU, and 3 eq. of DIPEA in DMF for 120 min. All dominant rotors were incorporated as the final residue in the sequence. **Cleavage from the solid support:** Once the desired linear sequence was synthesized, the terminal Fmoc-group was cleaved with 20 % piperidine in DMF *v/v* and the resin was thoroughly washed with CH_2Cl_2 (x3), DMF (x3), CH_2Cl_2 (x2). The resin was subsequently dried and treated with 30 % HFIP in CH_2Cl_2, five times for 10 min each to afford cleavage from the solid support. **Macrocyclisation:** The resulting peptide was dried thoroughly *in vacuo* before further use. In a flame dried RBF, 0.4-0.8 mmol of linear peptide obtained from SPPS was dissolved in THF (0.01 M). DEPBT (1.5 eq.) and DIPEA (6 eq.) were added to the solution. If the mixture remained cloudy, DMF stored under a nitrogen atmosphere was added dropwise, yielding a clear solution. The reaction mixture was left to stir overnight at room temperature (16 h). Tetraalkylammonium MP-carbonate resin (6 eq.) was then added to the reaction mixture and stirring was continued for an additional 24 h. The reaction was then filtered

through a solid-phase extraction vessel and rinsed with CH_2Cl_2 (2 mL). The crude filtrate was concentrated in vacuo and purified by reverse-phase C18 flash column chromatography.

1.3 Previously reported compounds

The following compounds were prepared according to literature procedures: 2-methyl-4H-benzo[d][1,3]oxazin-4-one, 2,5-dimethyl-4H-benzo[d][1,3]oxazin-4-one.[1]

The following compounds were previously reported by our lab: ***a***, ***b***, ***R*$_a$-1**, ***S*$_a$-1**, ***R*$_a$-2**, and ***S*$_a$-2**.[2]

1.4 Compound characterization

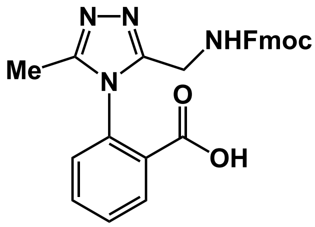

2-(3-((((9*H*-fluoren-9-yl)methoxy)carbonyl)amino)methyl)-5-methyl-4*H*-1,2,4-triazol-4-yl)benzoic acid (*a*) Synthesized according to general procedure in 1.2. White solid, 3.50 g, 7.7 mmol, 75% yield. ^{1}H NMR (500 MHz, DMSO-d_6) δ 13.15 (s, 1H), 8.03 (dd, J = 7.8, 1.7 Hz, 1H), 7.89 (d, J = 7.6 Hz, 2H), 7.73 (t, J = 6.0 Hz, 1H), 7.69 – 7.59 (m, 3H), 7.56 (dd, J = 7.7, 1.7 Hz, 1H), 7.47 – 7.37 (m, 4H), 7.33 (tt, J = 7.4, 1.4 Hz, 2H), 4.18 – 4.04 (m, 3H), 3.99 (d, J = 2.7 Hz, 1H), 2.03 (s, 3H). ^{13}C NMR (126 MHz, DMSO-d_6) δ 165.7, 155.6, 151.7, 151.4, 143.9, 143.7, 140.7, 133.1, 132.6, 131.5, 130.2, 129.7, 129.4, 127.7, 127.1, 125.4, 125.2, 120.1, 66.2, 46.5, 35.5, 10.4. HRMS (DART-TOF) [M+H$^+$] calculated for $C_{26}H_{23}N_4O_4$ = 455.1706, found = 455.1714.

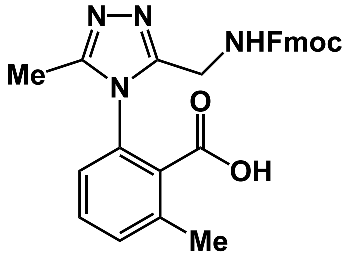

2-(3-(((((9*H*-fluoren-9-yl)methoxy)carbonyl)amino)methyl)-5-methyl-4*H*-1,2,4-triazol-4-yl)-6-methylbenzoic acid (*b*) Synthesized according to general procedure in 1.2. White solid, 2.46 g, 5.24 mmol, 83% yield; ^{1}H NMR (500 MHz, $CDCl_3$) δ 7.71 (d, *J* = 7.5 Hz, 2H), 7.57 (d, *J* = 7.5 Hz, 2H), 7.45 (d, *J* = 2.9 Hz, 2H), 7.34 (t, *J* = 7.5 Hz, 2H), 7.27 – 7.18 (m, 3H), 7.01 (s, 1H), 4.49 (dd, *J* = 17.1, 5.3 Hz, 1H), 4.40 – 4.32 (m, 1H), 4.28 – 4.17 (m, 2H), 4.13 (t, *J* = 7.5 Hz, 1H), 2.51 (s, 3H), 2.35 (s, 3H). ^{13}C NMR (126 MHz, $CDCl_3$) δ 169.0, 156.6, 153.7, 153.5, 144.0, 141.3, 138.5, 134.7, 133.5, 130.6, 128.7, 127.8, 127.2, 125.4, 124.9, 120.0, 67.4, 47.1, 36.1, 20.0, 10.4. HRMS (ESI+) [$M+H^{+}$] calculated for $C_{27}H_{25}N_4O_4$ = 469.1870, found = 469.1875.

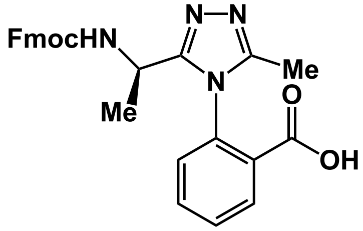

2-(3-((*R*)-1-((((9*H*-fluoren-9-yl)methoxy)carbonyl)amino)ethyl)-5-methyl-4*H*-1,2,4-triazol-4-yl)benzoic acid (*c*) Synthesized according to general procedure in 1.2. White solid, 1.03 g, 2.18 mmol, 43 % yield, 1:1.5 mixture of atropdiastereomers. ^{1}H NMR (500 MHz, DMSO-*d*$_6$) δ 13.09 (s, 1H), 8.07 – 7.99 (m, 1H), 7.88 (m, 3H), 7.77 – 7.51 (m, 3H), 7.50 – 7.27 (m, 5H), 4.66 – 4.38 (m, 1H), 4.13 – 3.85 (m, 3H), 2.03 (s, 2H), 1.38 (t, *J* = 7.1 Hz, 3H). ^{13}C NMR (126 MHz, DMSO-*d*$_6$) δ 165.6, 165.4, 155.1, 155.0, 154.8, 154.7, 151.6, 151.2, 144.0, 143.9, 143.7, 143.6, 140.7, 140.6, 133.5, 133.1, 133.1, 132.8, 131.7, 131.5, 130.1, 130.1, 130.1, 129.7, 129.0, 127.7, 127.7, 127.6, 127.1, 127.1, 127.0, 127.0, 125.5, 125.3, 120.1, 120.1, 120.1, 120.0, 65.7, 46.5, 46.5, 42.2, 42.0, 19.4, 18.9, 10.4, 10.4. HRMS (ESI+) calculated for $C_{27}H_{24}N_4O_4$ = 469.1870, found = 469.1869.

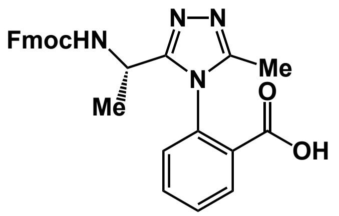

2-(3-((*S*)-1-((((9*H*-fluoren-9-yl)methoxy)carbonyl)amino)ethyl)-5-methyl-4*H*-1,2,4-triazol-4-yl)benzoic acid (*d*) Synthesized according to general procedure in 1.2. 0.85 g, 1.8 mmol, 51 % yield, 1: 1.4 mixture of atropdiastereomers. ^{1}H NMR (500 MHz, DMSO-*d*$_6$) δ 8.03 (ddd, *J* = 13.1, 7.8, 1.6 Hz, 1H), 7.93 – 7.83 (m, 3H), 7.77 – 7.53 (m, 4H), 7.53 – 7.27 (m, 7H), 4.62 – 4.39 (m, 1H), 4.30 – 3.86 (m, 3H), 2.03 (d, *J* = 2.2 Hz, 3H), 1.38 (t, *J* = 7.6 Hz, 3H). ^{13}C NMR (126 MHz, DMSO-*d*$_6$) δ 165.6, 165.4, 155.1, 154.9, 154.8, 154.7,

151.6, 151.2, 144.0, 143.9, 143.7, 143.6, 140.7, 140.6, 133.6, 133.1, 133.1, 132.78, 131.7, 131.5, 130.2, 130.1, 129.7, 129.0, 128.9, 127.7, 127.6, 127.6, 127.1, 127.1, 127.0, 125.5, 125.3, 120.1, 120.1, 120.1, 120.0, 65.7, 46.6, 46.5, 46.5, 42.2, 42.0, 19.4, 18.9, 10.4, 10.4. HRMS (ESI+) calculated for $C_{27}H_{24}N_4O_4$ = 469.1870, found = 469.1868.

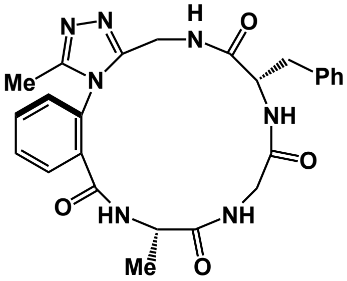

***cyclo*-[(*R**a*)-Trz(methyl)-Ala-Gly-Phe] (*R**a*-1)** Synthesized according to the procedure in 1.2. White solid, 44.5 mg, 0.091 mmol, 26% yield; ^{1}H NMR (500 MHz, DMSO-d_6) δ 9.20 (d, *J* = 5.2 Hz, 1H), 8.23 (dd, *J* = 7.9, 4.1 Hz, 1H), 8.06 (dd, *J* = 6.5, 2.6 Hz, 1H), 7.94 (dd, *J* = 6.0, 3.3 Hz, 1H), 7.86 (d, *J* = 8.6 Hz, 1H), 7.76 – 7.68 (m, 2H), 7.49 – 7.43 (m, 1H), 7.19 (m, 5H), 4.53 (dd, *J* = 14.8, 6.5 Hz, 1H), 4.19 (m, 1H), 4.01 (dd, *J* = 14.8, 7.9 Hz, 1H), 3.84 (m, 1H), 3.72 (dd, *J* = 14.8, 2.6 Hz, 1H), 3.18 (dd, *J* = 13.8, 4.5 Hz, 1H), 3.09 – 3.02 (m, 1H), 2.75 (dd, *J* = 13.8, 9.9 Hz, 1H), 2.04 (s, 3H), 1.18 (d, *J* = 6.9 Hz, 3H); ^{13}C NMR (101 MHz, DMSO-*d6*) δ 171.7, 169.6, 169.5, 165.8, 150.9, 150.9, 138.0, 132.8, 132.1, 131.2, 130.1, 130.0, 129.3, 129.0, 128.1, 126.2, 55.3, 49.9, 43.3, 36.9, 33.7, 15.6, 10.2; HRMS (ESI+) [M+H$^+$] calculated for $C_{25}H_{28}N_7O_4$ = 490.2197, found = 490.2204.

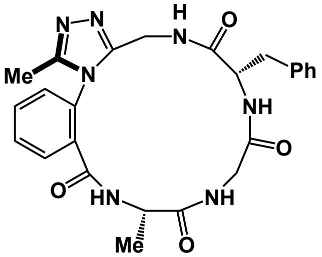

***cyclo*-[(*S**a*)-Trz(methyl)-Ala-Gly-Phe] (*S**a*-1)** Synthesized according to the procedure in 1.2. White solid, 41.1 mg, 0.084 mmol, 24% yield; White solid, 64 mg, 0.131 mmol, 28% yield; ^{1}H NMR (500 MHz, DMSO-d_6) δ 9.07 (d, *J* = 4.7 Hz, 1H), 8.73 (dd, *J* = 6.9, 5.4 Hz, 1H), 8.16 – 8.11 (m, 1H), 7.98 (dd, *J* = 5.9, 3.4 Hz, 1H), 7.79 – 7.72 (m, 2H), 7.49 – 7.43 (m, 1H), 7.38 (d, *J* = 8.4 Hz, 1H), 7.21 – 7.16 (m, 2H), 7.16 – 7.11 (m, 1H), 7.11 – 7.06 (m, 2H), 4.40 (td, *J* = 8.5, 5.3 Hz, 1H), 4.36 (dd, *J* = 15.0, 5.5 Hz, 1H), 4.09 (qd, *J* = 7.0, 4.6 Hz, 1H), 3.84 (dd, *J* = 15.0, 7.0 Hz, 1H), 3.77 (dd, *J* = 15.0, 4.1 Hz, 1H), 3.24 (dd, *J* = 15.0, 5.3 Hz, 1H), 2.95 (dd, *J* = 13.8, 5.3 Hz, 1H), 2.63 (dd, *J* = 13.7, 8.4 Hz, 1H), 1.98 (s, 3H), 1.30 (d, *J* = 7.0 Hz, 3H); ^{13}C NMR (101 MHz, DMSO-d_6) δ 173.4, 169.5, 169.5, 165.8, 151.1, 151.1, 137.5, 132.5, 132.1, 131.4, 130.2, 130.1 (overlap with 130.2), 129.7, 128.9, 128.0, 126.3, 54.3, 50.4, 43.6, 37.1, 32.4, 16.2, 10.4; HRMS (ESI+) [M+H$^+$] calculated for $C_{25}H_{28}N_7O_4$ = 490.2197, found = 490.2201.

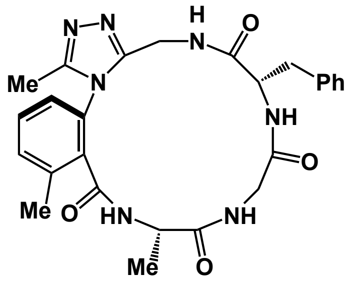

***cyclo*-[(*R*$_a$)-Trz(bis-methyl)-Ala-Gly-Phe] (*R*$_a$-2)** Synthesized according to the procedure in 1.2. White solid, 25 mg, 0.025 mmol, 20% yield; ^{1}H NMR (600 MHz, DMSO-d_6) δ 8.76 (d, J = 8.7 Hz, 1H), 8.72 (t, J = 6.2 Hz, 1H), 8.38 (d, J = 2.2 Hz, 1H), 7.66 (d, J = 8.2 Hz, 1H), 7.51 – 7.45 (m, 2H), 7.33 – 7.28 (m, 2H), 7.28 – 7.23 (m, 2H), 7.21 – 7.13 (m, 2H), 4.59 (dd, J = 14.8, 8.2 Hz, 1H), 4.26 (ddd, J = 11.8, 8.7, 2.9 Hz, 1H), 3.94 (qd, J = 7.0, 2.2 Hz, 1H), 3.79 (dd, J = 13.7, 6.2 Hz, 1H), 3.47 (d, J = 14.8 Hz, 1H), 3.32 – 3.28 (m, 1H), 3.07 (dd, J = 13.7, 6.2 Hz, 1H), 2.83 (dd, J = 14.0, 11.8 Hz, 1H), 2.32 (s, 3H), 2.23 (s, 3H), 1.08 (d, J = 7.0 Hz, 3H); ^{13}C NMR (126 MHz, DMSO-d_6) δ 173.4, 170.0, 169.4, 165.5, 151.0, 150.8, 139.0, 136.6, 135.6, 131.4, 129.8, 129.7, 129.0, 128.2, 126.3, 126.1, 55.2, 49.6, 43.7, 35.9, 33.4, 18.8, 16.6, 10.9; HRMS (ESI+) [M+H$^+$] calculated for $C_{26}H_{30}N_7O_4$ = 504.2354, found = 504.2350.

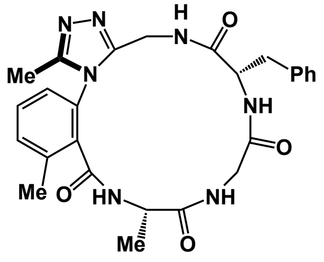

***cyclo*-[(*S*$_a$)-Trz(bis-methyl)-Ala-Gly-Phe] (*S*$_a$-2)** Synthesized according to the procedure in 1.2. White solid, 35 mg, 0.035 mmol, 28% yield; ^{1}H NMR (500 MHz, DMSO-d_6) δ 8.95 – 8.89 (m, 1H), 8.59 (d, J = 7.7 Hz, 1H), 8.18 (d, J = 9.1 Hz, 1H), 7.66 (dd, J = 7.0, 3.1 Hz, 1H), 7.57 – 7.44 (m, 2H), 7.32 – 7.20 (m, 5H), 7.20 – 7.14 (m, 1H), 4.55 – 4.50 (m, 1H), 4.47 (dd, J = 15.7, 7.2 Hz, 1H), 4.33 – 4.24 (m, 1H), 3.75 (dd, J = 14.4, 6.6 Hz, 1H), 3.69 (dd, J = 15.7, 3.1 Hz, 1H), 3.24 (dd, J = 14.4, 5.0 Hz, 1H), 3.20 (dd, J = 14.1, 4.1 Hz, 1H), 2.76 (dd, J = 14.1, 10.4 Hz, 1H), 2.39 (s, 3H), 2.06 (s, 3H), 1.27 (d, J = 6.8 Hz, 3H); ^{13}C NMR (126 MHz, DMSO-d_6) δ 173.1, 170.4, 169.3, 164.7, 151.8, 150.8, 138.4, 136.6, 135.8, 132.0, 130.1, 129.9, 128.9, 128.2, 126.2, 126.1, 54.2, 48.2, 44.0, 35.9, 33.8, 18.9, 17.9, 10.6; HRMS (ESI+) [M+H$^+$] calculated for $C_{26}H_{30}N_7O_4$ = 504.2354, found = 504.2349.

cyclo-[(S_a)-Trz(methyl/(R)-methyl)-Ala-Gly-Phe] **(S_a-3)**

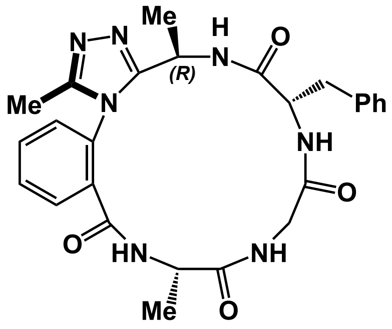

Synthesized according to the procedure in 1.2. White solid, 25 mg, 0.050 mmol, 31 % yield. Isolated as a single species. ^{1}H NMR (500 MHz, DMSO-d_6) δ 8.94 (d, J = 5.0 Hz, 1H), 8.70 (t, J = 6.3 Hz, 1H), 8.36 (d, J = 7.7 Hz, 1H), 8.10 – 8.03 (m, 1H), 7.77 – 7.69 (m, 2H), 7.47 – 7.41 (m, 1H), 7.24 – 7.12 (m, 3H), 6.84 (d, J = 7.4 Hz, 1H), 4.63 (p, J = 7.0 Hz, 1H), 4.29 (q, J = 7.3 Hz, 1H), 4.14 – 4.05 (m, 1H), 3.80 (dd, J = 15.6, 6.9 Hz, 1H), 3.33 – 3.25 (m, 3H), 2.71 (dd, J = 13.5, 7.4 Hz, 1H), 2.58 (dd, J = 13.5, 7.0 Hz, 1H), 1.99 (s, 3H), 1.30 (dd, J = 7.0, 4.4 Hz, 6H). ^{13}C NMR (126 MHz, DMSO-d_6) δ 173.4, 169.5, 168.5, 165.5, 155.1, 151.0, 137.5, 132.6, 132.5, 131.5, 130.4, 130.0, 129.5, 129.1, 128.1, 126.3, 54.3, 50.6, 43.5, 19.2, 16.3, 10.5. HRMS (DART+) [M+H$^+$] calculated for $C_{26}H_{30}N_7O_4$ = 504.23538, found = 504.23551.

cyclo-[(R_a)-Trz(methyl/(S)-methyl)-Ala-Gly-Phe] **(R_a-4)**

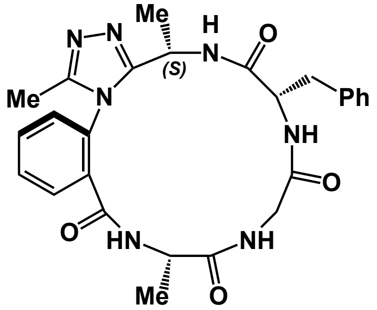

Synthesized according to the procedure in 1.2. White solid, 35 mg, 0.070 mmol, 11 % yield. ^{1}H NMR (500 MHz, DMSO-d_6) δ 9.47 (d, J = 2.7 Hz, 1H), 8.83 (dd, J = 11.6, 5.2 Hz, 2H), 7.94 (dd, J = 7.7, 1.7 Hz, 1H), 7.84 – 7.71 (m, 2H), 7.52 (dd, J = 7.9, 1.3 Hz, 1H), 7.36 (d, J = 9.1 Hz, 1H), 7.09 – 6.93 (m, 5H), 5.20 – 5.11 (m, 1H), 4.46 (ddd, J = 9.2, 6.7, 4.7 Hz, 1H), 4.03 (dd, J = 15.9, 7.7 Hz, 1H), 3.96 (dd, J = 7.0, 2.7 Hz, 1H), 3.26 (dd, J = 15.8, 4.8 Hz, 1H), 2.83 (dd, J = 13.6, 4.8 Hz, 1H), 2.70 (dd, J = 13.6, 6.8 Hz, 1H), 1.88 (s, 3H), 1.30 (d, J = 7.0 Hz, 3H), 0.86 (d, J = 7.2 Hz, 3H). ^{13}C NMR (126 MHz, DMSO-d_6) δ 173.3, 169.4, 168.1, 167.0, 153.3, 151.9, 136.2, 132.8, 132.5, 131.1, 130.6, 130.4, 129.3, 127.9, 126.5, 55.3, 51.4, 43.5, 43.2, 38.5, 18.1, 15.7, 10.2. HRMS (DART+) [M+H$^+$] calculated for $C_{26}H_{30}N_7O_4$ = 504.23538, found = 504.23547.

1.5 Variable-Temperature NMR

Variable-temperature NMR spectra of **R_a-1**, **S_a-1**, **R_a-2**, and **S_a-2** was previously reported. The data has been reproduced here for clarity.[2]

VT-NMR of R_a-1 (25 → 50 °C)

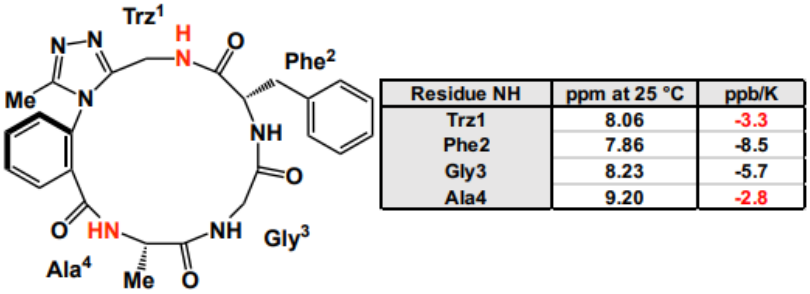

Residue NH	ppm at 25 °C	ppb/K
Trz1	8.06	-3.3
Phe2	7.86	-8.5
Gly3	8.23	-5.7
Ala4	9.20	-2.8

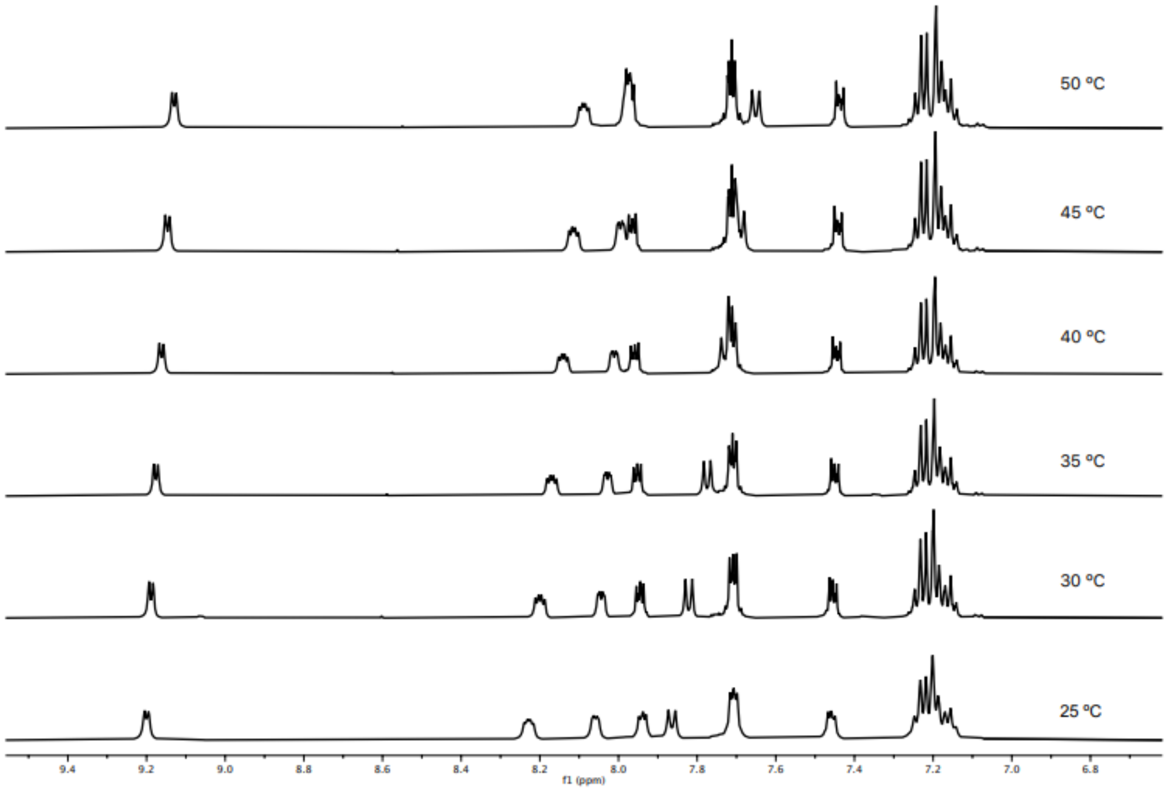

VT-NMR of S_a-1 (25 → 50 °C)

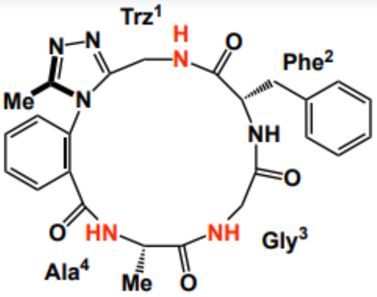

Residue NH	ppm at 25 °C	ppb/K
Trz1	8.13	-3.2
Phe2	7.38	-5.2
Gly3	8.73	-3.7
Ala4	9.07	-3.9

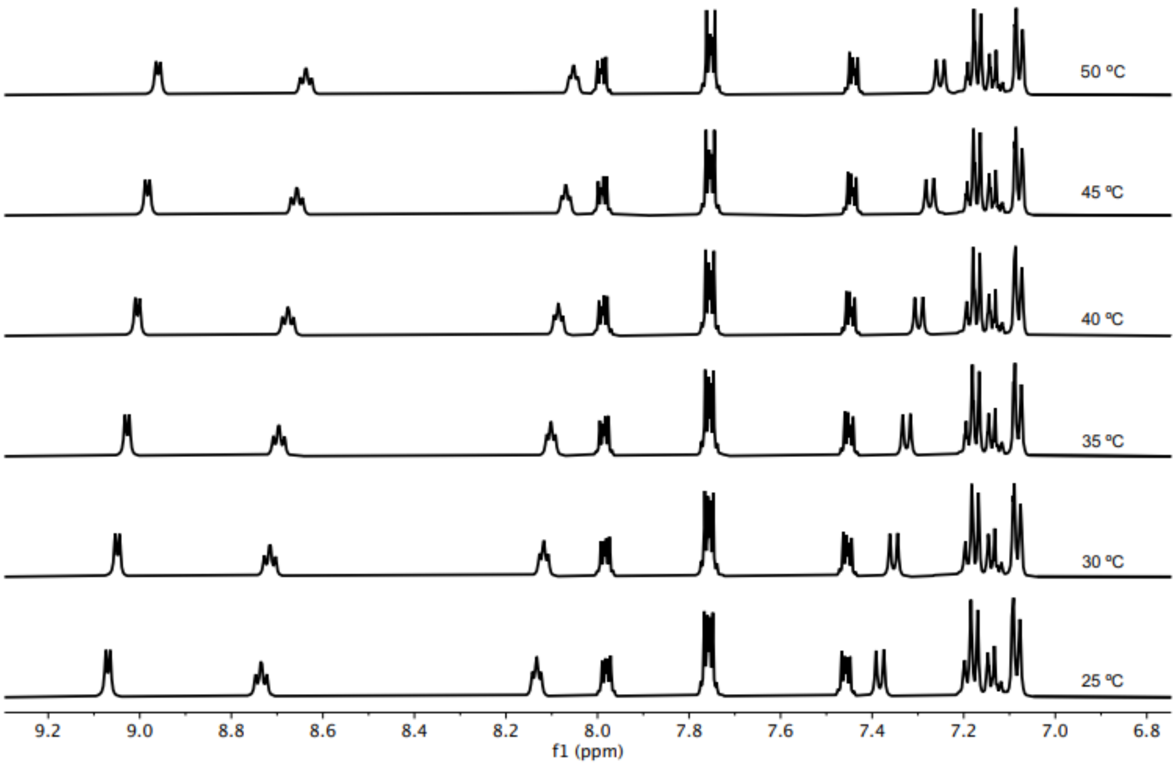

VT-NMR of R_a-2 (25 → 55 °C)

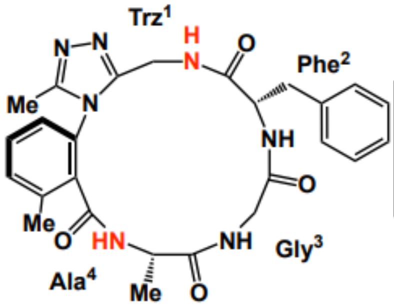

Residue NH	ppm at 25 °C	ppb/K
Trz1	7.66	-2.83
Phe2	8.76	-11.03
Gly3	8.73	-7.17
Ala4	8.38	-3.17

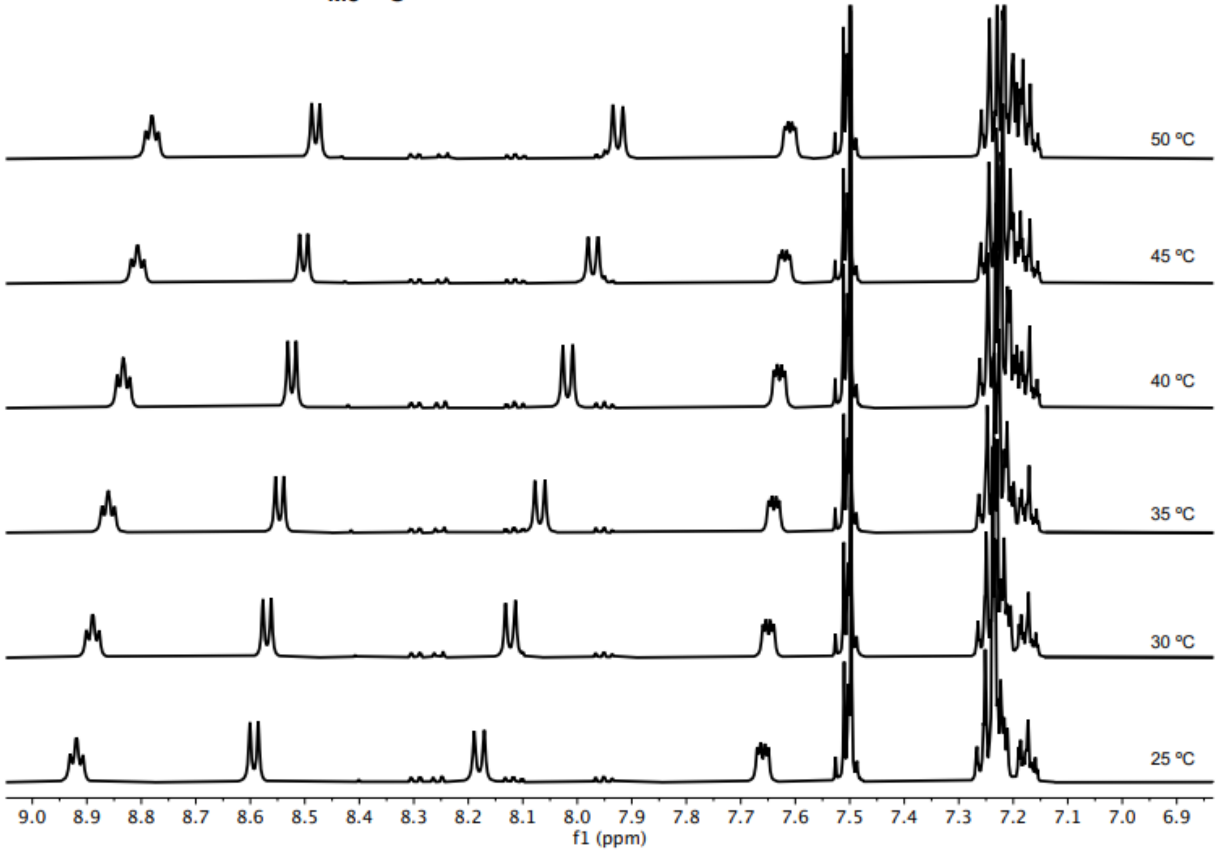

VT-NMR of S_a-2 (25 → 55 °C)

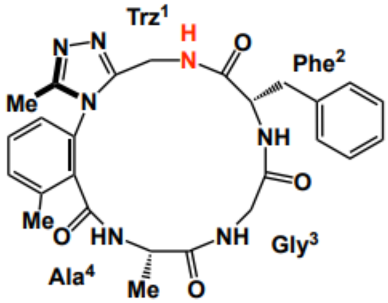

Residue NH	ppm at 25 °C	ppb/K
Trz1	7.66	-2.0
Phe2	8.18	-10.0
Gly3	8.92	-5.54
Ala4	8.59	-4.51

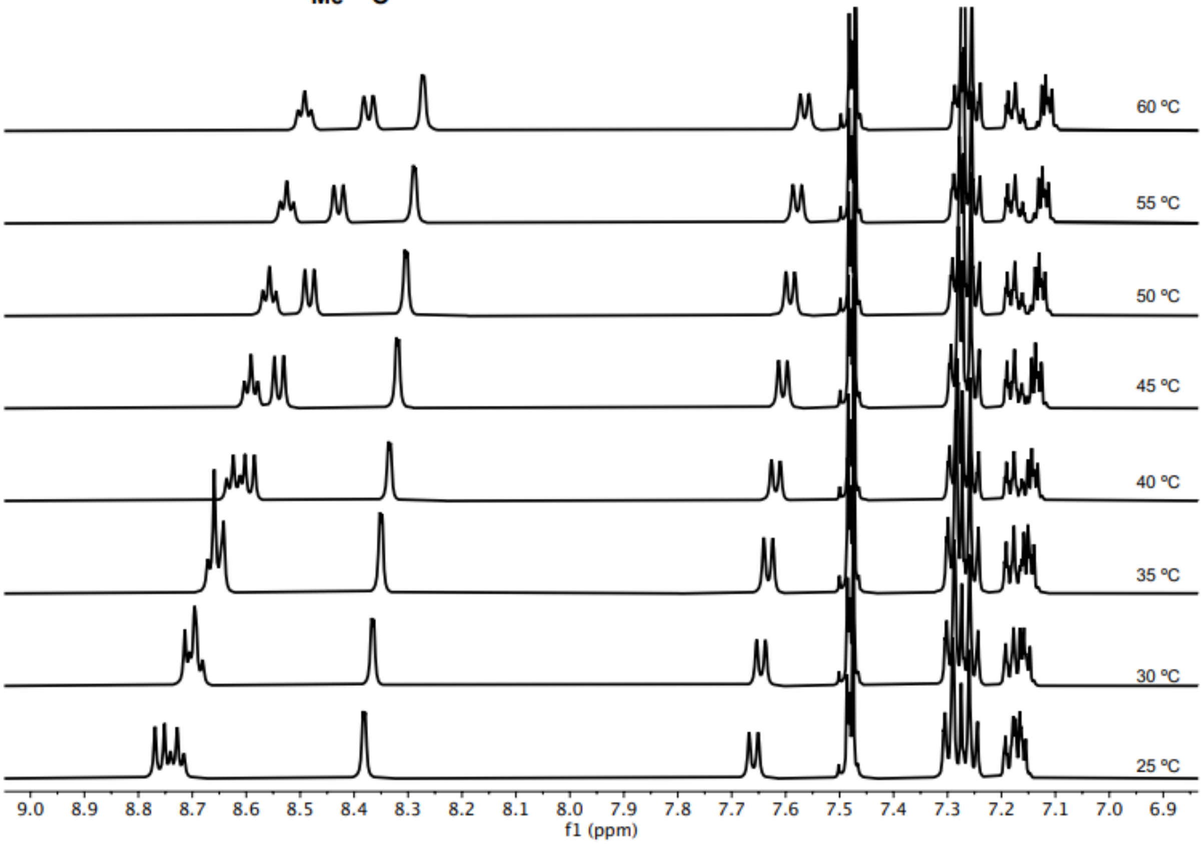

VT-NMR of S_a-3 (25 → 55 °C)

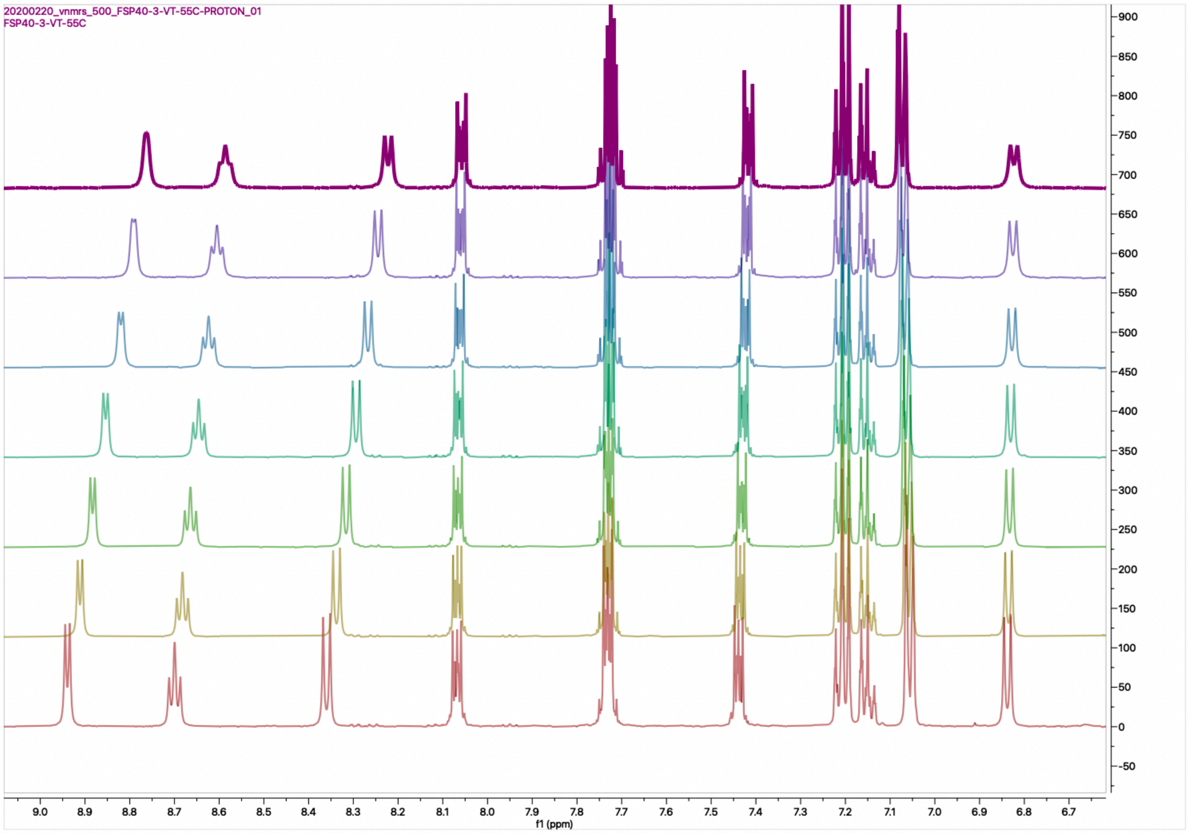

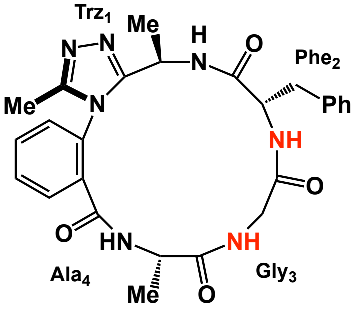

(S_a)-3
ppm at 25 °C; $^3J_{HNCH}$; ppb/K

Trz$_1$: 8.36 ppm; 7.74 Hz; **-4.57 ppb/K**
Phe$_2$: 6.84 ppm; 7.43 Hz; **-0.49 ppb/K**
Gly$_3$: 8.73 ppm; **-3.78 ppb/K**
Ala$_4$: 8.94 ppm; 5.04 Hz; **-5.88 ppb/K**

VT-NMR of R_a-4 (25 → 55 °C)

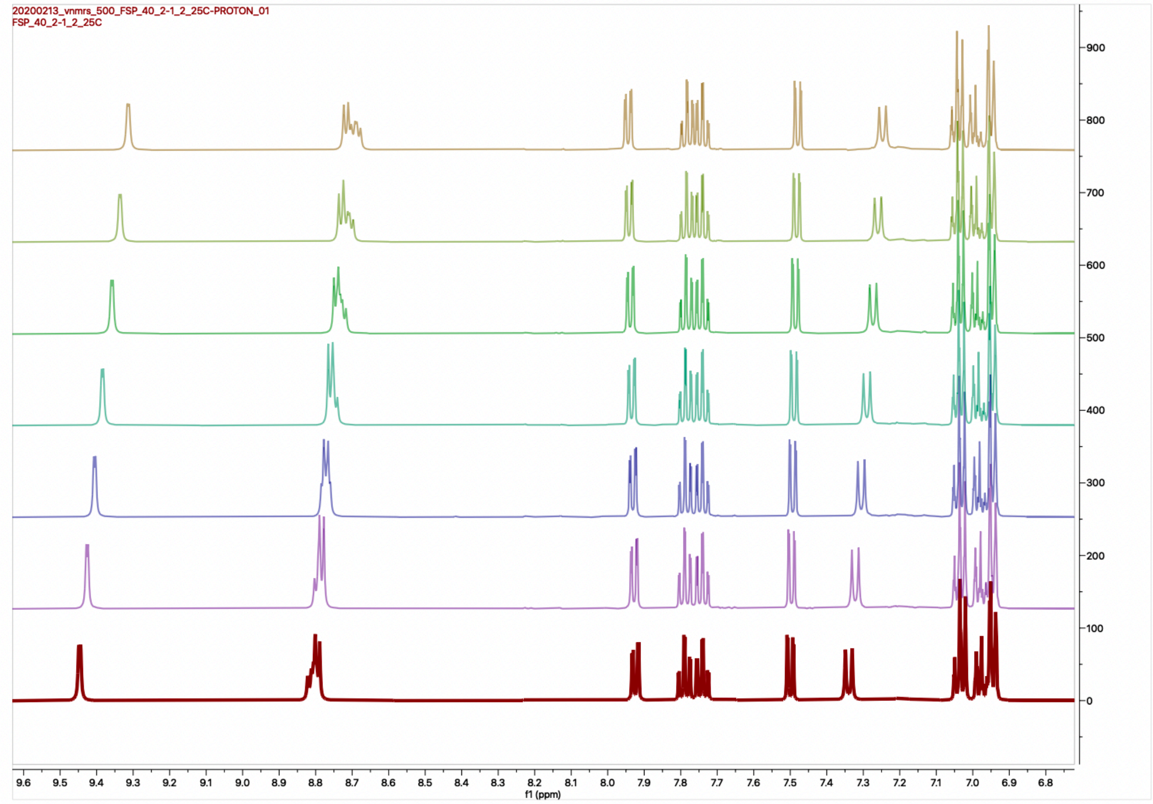

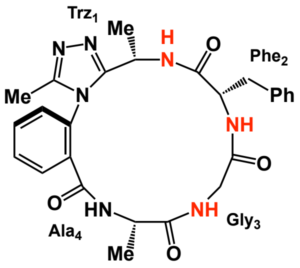

(R_a)-4
ppm @ 25 °C; $^3J_{HNCH}$; ppb/K

Trz$_1$: 8.81 ppm; overlap; **-2.62 ppb/K**
Phe$_2$: 7.36 ppm; 9.12 Hz; **-3.10 ppb/K**
Gly$_3$: 8.83 ppm; **-4.00 ppb/K**
Ala$_4$: 9.47 ppm; 2.72 Hz; **-4.43 ppb/K**

1.6 Molecular dynamics-derived structure determination

The NMR structures were determined by a combination of NMR derived distance information and molecular dynamics studies. ROESY spectra were integrated by using MestreNova (v. 10.0.2, Mestrelab Research S.L.) software. Integrated volumes of ROE crosspeaks were converted to proton interatomic distances using an inverse sixth power relationship. A reference integral was calculated as the average integral between sets of geminal protons which was then set to the calculated geminal interproton distance of 1.78 Å. The calculated distances were adjusted upwards and downwards by 10% to give upper and lower bounds to account for uncertainty in interproton distances.^{3}J coupling constants were recorded from the 1H spectrum. NH-CαH ^{3}J coupling constants of < 6 Hz were assigned phi dihedral values of -60° +/- 25°. NH- CαH 3J coupling constants of > 8 Hz were assigned phi values of -120° +/- 25°. Crude structures of macrocycles were generated by a restrained Monte Carlo low mode molecular mechanics conformational search with an implicit solvent model (DMSO) in Macromodel (Schrodinger LLC, v11.0). The 3D structure was restrained using flat-bottomed potential wells of 1000 kJ/mol with a well width defined by the upper and lower bound pairwise of the internuclei distances. The simulation was carried out for 100 steps of sampling per rotatable bond in the structure. The dihedral values of phi angles were harmonically constrained in potential wells of mean values of -60° +/- 25° for NH-CαH ^{3}J coupling constants of < 6 Hz and -120° +/- 25° for NH-CαH ^{3}J coupling constants of > 8 Hz. The output structures were ranked by energy. The structures were then checked for violations of the experimental distance and dihedral restraints. The lowest energy structure which satisfied these tests was passed for molecular dynamics study as the representative study. Solvent explicit molecular dynamics simulations were carried out with the Desmond Molecular Dynamics software module (D.E. Shaw, v4.4) running inside Maestro (Schrodinger LLC, v2015- 2). The OPLS4 force

field was used for parameterization of the peptidomimetic macrocycle. The macrocycle representative structure was placed in an octahedral box solvent box (DMSO) with a minimum distance of 12 Å between solute atoms and the box boundary. The solvated box was minimized then brought to 300 K from 10 K using a restrained dynamics regime. Coulombic interactions were grouped into near- and far interactions with a near-interaction cutoff of 9 Å. Bonds were constrained with the SHAKE algorithm and an integration time step of 2 fs was used. The final MD production run was 100 ns in length with energy value recording every 1.2 ps and trajectory recording every 4.8 ps. The run trajectory was clustered using the Trajectory Clustering script within Maestro with a 0.4 Å RMSD cutoff for variation between backbone heavy atoms and a sampling frequency of 10%. The most populated cluster was taken as the "preferred" structure i.e. that conformation which the molecule spent most time in the dynamics run. This process was repeated for each potential diastereomer to assign the stereochemistry of the new sp^3 center in the linker. The distances from the most populated cluster for each diastereomer were then measured and compared with the initial NOE derived distances. The simulation distances for both diastereomers is included in the individual entry for each compound. The cluster that displayed the least violations was selected as the probable species isolated.

1.7 NOE-derived distance restraints and calculated distances

NOE tables for molecular dynamics simulations of ***R_a*-1**, ***S_a*-1**, ***R_a*-2**, and ***S_a*-2** were previously reported and are provided here for clarity.[2] NOE tables for molecular dynamics simulations of ***S_a*-3** and ***R_a*-4** are also included in this section.

R_a-1

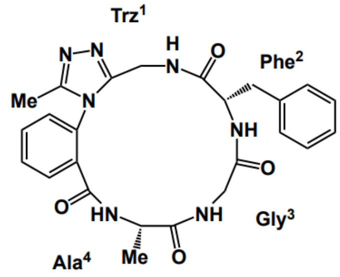

$^{3}J_{HNCH}$

Phe^2 = 7.86 (d, J = 8.6 Hz, 1H)

Ala^4 = 9.20 (d, J = 5.3 Hz, 1H)

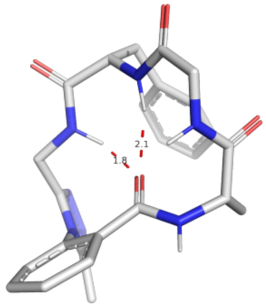

Number	Residue 1	Atom 1	Residue 2	Atom 2	Calculated NOE (Å)	NOE Lower Bound (Å)	NOE Upper Bound (Å)	MD Average Distance (Å)	Violation (Å)
1	Ala4	HN	Trz1	Ar-CH_3	3.80	3.42	4.18	5.1	-0.92
2	Ala4	HN	Ala4	Hα	2.37	2.14	2.61	2.2	0.00
3	Ala4	HN	Trz1	Ar-CH	2.37	2.14	2.61	2.0	0.14
4	Ala4	HN	Gly3	HN	3.80	3.42	4.18	3.0	0.42
5	Gly3	HN	Ala4	Hα	2.45	2.21	2.70	3.0	-0.30
6	Gly3	HN	Phe2	HN	3.39	3.05	3.73	2.4	0.65
7	Gly3	HN	Trz1	Ar-CH	3.39	3.05	3.73	5.0	-1.27
8	Trz1	HN	Phe2	Hα	3.02	2.72	3.32	3.6	-0.28
9	Trz1	HN	Phe2	HN	2.64	2.37	2.90	2.1	0.27
10	Phe2	HN	Phe2	Hα	2.82	2.54	3.10	2.9	0.00

S_a-1

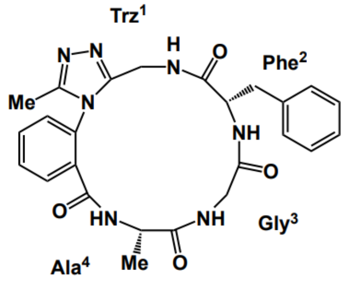

$^{3}J_{HNCH}$

Phe^2 = 7.38 (d, J = 8.4 Hz, 1H)

Ala^4 = 9.07 (d, J = 4.7 Hz, 1H)

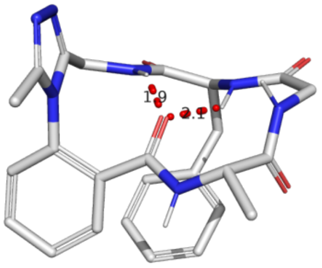

Number	Residue 1	Atom 1	Residue 2	Atom 2	Calculated NOE (Å)	NOE Lower Bound (Å)	NOE Upper Bound (Å)	MD Average Distance (Å)	Violation (Å)
1	Ala4	HN	Ala4	Hα	2.85	2.57	3.14	2.8	0.00
2	Ala4	HN	Trz1	Ar-CH	2.25	2.02	2.47	2.5	-0.03
3	Ala4	HN	Gly3	HN	3.85	3.46	4.23	4.3	-0.07
4	Gly3	HN	Ala4	Hα	2.25	2.02	2.47	2.3	0.00
5	Gly3	HN	Phe2	HN	3.43	3.08	3.77	2.4	0.68
6	Trz1	HN	Phe2	Hα	2.58	2.32	2.84	3.3	-0.46
7	Trz1	HN	Phe2	HN	2.78	2.50	3.06	1.6	0.90
8	Phe2	HN	Phe2	Hα	3.05	2.75	3.36	2.9	0.00

R$_a$-2

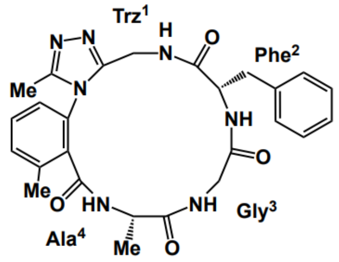

$^3J_{HNCH}$

Phe^2 = 8.76 (d, J = 8.7 Hz, 1H)

Ala^4 = 8.38 (d, J = 2.3 Hz, 1H)

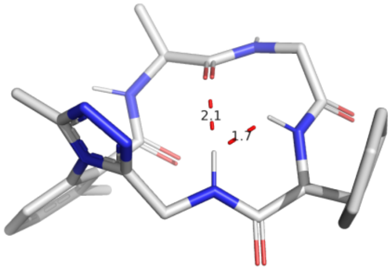

Number	Residue 1	Atom 1	Residue 2	Atom 2	Calculated NOE (Å)	NOE Lower Bound (Å)	NOE Upper Bound (Å)	MD Average Distance (Å)	Violation (Å)
1	Phe2	HN	Trz1	HN	2.38	2.14	2.62	1.7	0.44
2	Gly3	HN	Ala4	Hα	2.22	2.00	2.45	2.3	0.00
3	Ala4	HN	Trz1	CH3	3.14	2.83	3.46	3.2	0.00
4	Ala4	HN	Trz1	Ar-CH3	3.36	3.03	3.70	3.9	-0.20
5	Ala4	HN	Ala4	Hα	2.80	2.52	3.08	2.8	0.00
6	Trz1	HN	Phe2	Hα	3.14	2.83	3.46	3.2	0.00

S$_a$-2

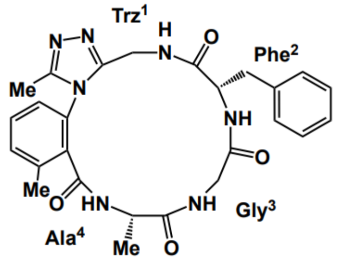

$^3J_{HNCH}$

Phe^2 = 8.18 (d, J = 9.1 Hz, 1H)

Ala^4 = 8.59 (d, J = 7.7 Hz, 1H)

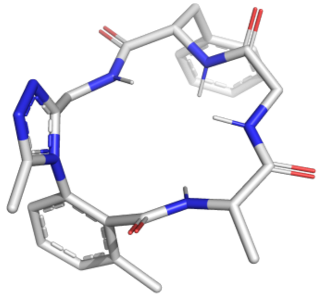

Number	Residue 1	Atom 1	Residue 2	Atom 2	Calculated NOE (Å)	NOE Lower Bound (Å)	NOE Upper Bound (Å)	MD Average Distance (Å)	Violation (Å)
1	Gly3	HN	Ala4	Hα	2.44	2.19	2.68	2.9	-0.22
2	Gly3	HN	Phe2	HN	3.41	3.07	3.75	2.2	0.87
3	Ala4	HN	Trz1	Trz-CH_3	3.83	3.45	4.21	5.5	-1.29
4	Ala4	HN	Trz1	ArCH_3	3.19	2.87	3.51	3.1	0.00
5	Ala4	HN	Ala4	Hα	2.77	2.49	3.04	2.9	0.00
6	Ala4	HN	Trz1	HN	3.83	3.45	4.21	3.4	0.05
7	Phe2	HN	Trz1	HN	2.61	2.35	2.87	2.4	0.00
8	Trz1	HN	Phe2	Hα	3.04	2.73	3.34	2.8	0.00

S$_a$-3

Number	Residue 1	Atom	Residue 2	Atom	Calculated NOE (Å)	NOE upper bound (Å)	NOE lower bound (Å)	MD distance (Å)	Violation (Å)
1	Ala1	NH	DR-aryl	6-CH	0.16	2.64	2.16	2.08	-0.08
2	Ala1	NH	Ala1	CHα	0.03	3.49	2.85	2.97	0
3	Gly2	NH	Ala1	NH	0.01	4.19	3.43	3.32	-0.11
4	Gly2	NH	Ala1	CHα	0.19	2.56	2.1	2.74	0.18
5	Gly2	NH	Phe3	NH	0.01	4.19	3.43	4.01	0
6	Phe3	NH	Phe3	CHα	0.03	3.49	2.85	2.82	0
7	Triazole	NH	Phe3	NH	0.01	4.19	3.43	3.57	0
8	Triazole	NH	Phe3	CHα	0.12	2.77	2.26	2.46	0
9	Triazole	NH	Triazole	CHα	0.03	3.49	2.85	2.66	-0.19
10	Triazole	CHα	DR-aryl	3-CH	0.05	3.2	2.62	3.16	0

***Ra*-4**

Number	Residue 1	Atom	Residue 2	Atom	Calculated NOE (Å)	NOE upper bound (Å)	NOE lower bound (Å)	MD distance (Å)	Violation (Å)
1	Ala1	NH	Ala1	CHα	0.04	3.32	2.72	2	-0.72
2	Ala1	NH	DR-aryl	6-CH	0.12	2.77	2.26	2.2	-0.06
3	Gly2	NH	Ala1	CHα	0.13	2.73	2.23	3.2	0.47
4	Phe3	NH	Phe3	CHα	0.02	3.73	3.05	2.9	-0.15
5	Triazole	NH	Phe3	NH	0.11	2.81	2.3	2	-0.3

1.8 Measurement of φ / ψ torsion angles

φ / ψ torsion angles were extracted from the most populated MD clusters using PyMOL and are tabulated below. CIF structure files for cyclo(PGLDT) odz/Et **5** and cyclo(PLDTG) odz/Et **6** were obtained from the supplementary materials of previously reported molecular dynamics simulations.[3]

***Ra*-1**	**Ala1**		**Gly2**		**Phe3**		**Triazole**	
	**φ (°)**	**ψ (°)**	**φ (°)**	**ψ (°)**	**φ (°)**	**ψ (°)**	**φ (°)**	**ψ (°)**
**MD Cluster 1**	70.03	14.474	96.852	-8.085	-126.686	-28.725	129.574	89.504
**MD Cluster 2**	70.972	16.235	90.745	3.412	-145.351	-21.928	129.535	96.629
**MD Cluster 3**	-151.146	25.8	83.826	-4.626	-151.661	22.433	94.721	112.149
**MD Cluster 4**	74.472	8.809	93.221	-7.331	-133.719	-1.6	101.387	85.013
**MD Cluster 5**	75.062	-0.779	112.851	7.867	-143.266	-29.934	134.619	102.528
**MD Cluster 6**	59.313	14.599	98.962	-3.152	-137.398	-7.181	99.92	92.302
**MD Cluster 7**	72.521	23.77	100.093	-3.653	-147.199	-52.741	161.133	83.938
**MD Cluster 8**	70.56	-4.003	114.162	1.179	-145.352	3.93	92.304	101.762
**MD Cluster 9**	70.725	4.898	96.469	3.047	-133.795	1.721	102.598	93.917
**MD Cluster 10**	53.515	20.778	92.888	6.868	-139.495	-48.638	150.33	93.671
**MD Cluster 11**	53.509	20.782	92.884	6.873	-139.498	-48.639	150.33	93.674
**MD Cluster 12**	70.722	4.898	96.471	3.048	-133.794	1.719	102.594	93.915
**MD Cluster 13**	70.557	-4.004	114.16	1.183	-145.351	3.927	92.307	101.764
**MD Cluster 14**	72.52	23.773	100.092	-3.655	-147.201	-52.74	161.127	83.934
**MD Cluster 15**	59.317	14.593	98.967	-3.156	-137.392	-7.19	99.919	92.307

Sa -1	Ala1		Gly2		Phe3		Triazole	
	φ (°)	ψ (°)	φ (°)	ψ (°)	φ (°)	ψ (°)	φ (°)	ψ (°)
**MD Cluster 1**	-95.132	164.264	45.545	-86.406	-95.275	-10.322	-97.763	-139.432
**MD Cluster 2**	-58.349	102.102	65.145	17.368	-152.039	7.465	168.869	-67.955
**MD Cluster 3**	-85.29	175.322	59.679	-69.75	-143.773	16.418	-94.956	-134.97
**MD Cluster 4**	-60.027	99.585	82.716	11.737	-135.845	-31.576	-147	-86.969
**MD Cluster 5**	-56.145	104.762	85.207	3.743	-159.814	4.328	176.181	-81.827
**MD Cluster 6**	-56.084	87.26	95.855	8.498	-156.06	-0.491	179.571	-83.237
**MD Cluster 7**	-66.556	113.185	88.673	-1.244	-153.499	-18.081	-167.87	-86.473
**MD Cluster 8**	-51.059	107.649	97.213	-2.91	-152.993	-33.153	-147.632	-81.782
**MD Cluster 9**	-63.324	103.564	89.812	0.095	-148.241	4.641	172.064	-75.183
**MD Cluster 10**	-54.403	110.807	82.043	4.873	-151.788	-12.718	-165.149	-94.13
**MD Cluster 11**	-56.084	87.26	95.855	8.498	-150.06	-0.491	-179.571	-83.237
**MD Cluster 12**	-66.556	113.185	88.673	-1.244	-153.499	-18.081	-167.87	-86.473
**MD Cluster 13**	-51.059	107.649	97.213	-2.91	-152.993	-33.153	-147.632	-81.782
**MD Cluster 14**	-63.324	103.564	89.812	0.095	-148.241	4.641	-172.064	-75.183
**MD Cluster 15**	-54.403	110.807	82.043	4.873	-151.788	-12.718	-165.149	-94.13

Ra -2	Ala1		Gly2		Phe3		Triazole	
	φ (°)	ψ (°)	φ (°)	ψ (°)	φ (°)	ψ (°)	φ (°)	ψ (°)
**MD Cluster 1**	-58.229	114.858	72.993	-90.036	-124.142	-3.204	152.034	126.639
**MD Cluster 2**	-55.391	113.609	67.481	-62.629	-146.843	0.246	-179.937	116.753
**MD Cluster 3**	-54.469	133.946	62.788	-61.848	-141.609	10.05	145.951	121.263
**MD Cluster 4**	-64.406	120.475	82.329	-66.854	-143.949	-5.345	158.756	104.17
**MD Cluster 5**	-47.37	130.356	71.853	-64.316	-149.184	1.685	162.295	115.82
**MD Cluster 6**	-55.126	131.088	81.387	-80.075	-150.948	9.106	162.319	101.113
**MD Cluster 7**	-61.282	157.314	74.256	-76.656	-153.195	11.427	148.723	115.647
**MD Cluster 8**	-62.112	152.221	65.174	-88.033	-141.572	9.071	179.555	111.209
**MD Cluster 9**	-53.707	140.235	74.412	-76.5	-153.254	19.221	158.185	107.778
**MD Cluster 10**	-45.429	142.528	75.179	-69.361	-155.346	3.161	170.978	122.661
**MD Cluster 11**	-45.417	142.524	75.178	-69.365	-155.341	3.16	170.981	122.163
**MD Cluster 12**	-53.713	140.235	74.413	-76.559	-153.259	19.224	158.186	107.777
**MD Cluster 13**	-62.114	152.215	65.176	-88.027	-141.576	9.071	179.553	111.212
**MD Cluster 14**	-61.286	157.315	74.257	-76.654	-153.195	11.423	148.724	115.644
**MD Cluster 15**	-55.125	131.091	81.387	-80.076	-150.95	9.108	162.315	101.112

Sa-2	Ala1		Gly2		Phe3		Triazole	
	φ (°)	ψ (°)	φ (°)	ψ (°)	φ (°)	ψ (°)	φ (°)	ψ (°)
**MD Cluster 1**	-148.813	-5.169	-131.075	-41.837	-158.247	32.933	-144.462	-105.009
**MD Cluster 2**	-156.438	66.815	149.217	-31.217	-154.853	40.369	-159.827	-108.585
**MD Cluster 3**	-146.049	-11.446	-103.09	-40.611	-162.917	25.38	-127.037	-112.784
**MD Cluster 4**	-153.799	72.081	159.228	-63.321	-158.764	31.818	-145.992	-121.284
**MD Cluster 5**	-133.334	-17.61	-93.376	-54.919	-165.946	44.82	-140.85	-110.995
**MD Cluster 6**	-147.814	53.269	156.228	-33.228	-148.326	19.033	-139.939	-115.983
**MD Cluster 7**	-148.163	22.92	168.625	-21.027	-144.177	26.61	-155.9	-104.277
**MD Cluster 8**	-160.905	51.058	171.827	-38.527	-151.352	30.623	-143.551	-125.905
**MD Cluster 9**	-156.365	13.563	-142.085	-49.799	-146.565	23.457	-146.016	-105.685
**MD Cluster 10**	-158.73	31.762	-172.552	-46.062	-154.595	35.457	-152.518	-107.812
**MD Cluster 11**	-158.727	31.765	-172.552	-46.068	-154.589	34.454	-152.519	-107.813
**MD Cluster 12**	-156.363	13.565	-142.083	-49.804	-146.563	23.458	-146.015	-105.687
**MD Cluster 13**	-160.902	51.059	171.824	-38.53	-151.35	30.631	-143.555	-125.908
**MD Cluster 14**	-148.162	22.922	168.629	-21.028	-147.18	28.609	-155.898	-104.275
**MD Cluster 15**	-147.811	53.272	156.229	-33.228	-148.32	19.029	-139.938	-115.98

Sa-3	Ala1		Gly2		Phe3		Triazole		Biaryl	Benzamide
	φ (°)	ψ (°)	φ (°)	ψ (°)	φ (°)	ψ (°)	φ (°)	ψ (°)	θ (°)	θ (°)
**MD Cluster 1**	-75.8	40.5	176.5	-145	-69	73.1	173.5	-129.6	105	-163.2
**MD Cluster 2**	-79.6	55.9	155	-135.4	-70.8	64.1	-173.2	-122.7	105	-162
**MD Cluster 3**	-80.6	35.7	163.4	-128.9	-65.9	67.9	170.5	-122.3	114.9	-147.5
**MD Cluster 4**	-96.5	51.1	173.4	-140.7	-69.9	68.8	173.6	-126.7	109.5	-152.3
**MD Cluster 5**	-79.1	43.1	157.8	-119.6	-72.2	72	173	-121.7	92.3	-164.7
**MD Cluster 6**	-67.3	28.7	172.2	-145.1	-57.9	77.7	163.9	-114.1	114.9	-163.9
**MD Cluster 7**	-77.7	40.8	162	-128.1	-59.4	71.5	166.7	-112.9	113.9	-158.9
**MD Cluster 8**	-84.3	46.6	148.3	-121.2	-68.2	70.2	167	-120.6	106.3	-153.9
**MD Cluster 9**	-80.3	33.7	-171.9	-131.1	-77.4	85.5	168.4	-118.9	105.4	-168.6
**MD Cluster 10**	-83.9	48.2	155.6	-131.4	-70.9	68	172	-119.8	114	-150.4

Ra-4	Ala1		Gly2		Phe3		Triazole		Biaryl	Benzamide
	φ (°)	ψ (°)	φ (°)	ψ (°)	φ (°)	ψ (°)	φ (°)	ψ (°)	θ (°)	θ (°)
**MD Cluster 1**	66.7	10.1	94.8	-2.1	-119.6	-6.6	70.7	99.6	-80.6	-155.5
**MD Cluster 2**	49.5	17.7	98.6	14.3	-145.7	-68.9	176.4	104.3	-86.7	-139.3
**MD Cluster 3**	55.9	25.4	80.6	-13.2	-110.2	4.1	78.7	97.7	-88.5	-158.6
**MD Cluster 4**	57.1	19.2	109.5	5.9	-147.5	-72.6	-177	98.4	-95.4	-148
**MD Cluster 5**	66.2	5.6	96.7	-5	-105.3	-16.2	76.2	109.4	-75	-153.6
**MD Cluster 6**	65.3	15.4	90.9	9.1	-137.3	-66.3	174.1	93.1	-92.4	-146.7
**MD Cluster 7**	64	23.7	90.7	-4.1	-100.8	-13.9	70.1	101.1	-85.8	-169.4
**MD Cluster 8**	61.3	12.2	100.6	-3.3	-125.5	10	74.9	91.8	-94.9	-158.4
**MD Cluster 9**	51.3	27	84.8	11.7	-139	-64.6	174.5	99.4	-89.6	-141.1
**MD Cluster 10**	57.7	20.7	88.1	1.3	-125.2	-6.3	79.4	95.7	-90	-153.6

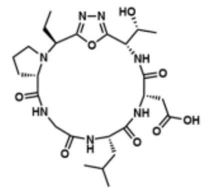

cyclo(PGLDT) odz/Et 5		
	φ (°)	ψ (°)
**Pro1**	-95.40	16.00
**Gly2**	-126.80	151.20
**Leu3**	-42.40	139.60
**Asp4**	73.50	-39.70
**Thr5**	-85.10	-64.80
**Odz6**	-178.40	-58.10

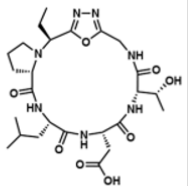

cyclo(PLDTG) odz/Et 6		
	φ (°)	ψ (°)
**Pro1**	-91.70	-11.20
**Leu2**	-86.50	173.90
**Asp3**	-65.80	109.60
**Thr4**	56.90	13.70
**Gly5**	-105.40	-53.10
**Odz6**	-177.50	-66.80

1.9 Van't Hoff analysis of dominant rotor macrocycles

The Van't Hoff analysis for ***R**$_a$***-1**/***S**$_a$***-1** and ***R**$_a$***-2**/***S**$_a$***-2** has been reported previously but the data and methods are also presented here for clarity.[2] This analysis was not carried out for single-welled system ***S**$_a$***-3** or ***R**$_a$***-4**, which was isolated as a single atropdiastereomer amongst three other minor species.

General procedure for measuring equilibrium constants: A 1-dram oven-dried vial containing ~3.5 milligrams of macrocycle was dissolved in 0.25 mL of DMSO-d_6 using a 1 mL syringe and transferred into a three-millimeter NMR tube. The samples were heated for 24 h to each specified using an oil bath controlled by an IKA programmable temperature probe. After 24 h, the sample was removed and immediately submerged in a dry ice/isopropanol bath for 1 minute. The sample was left to thaw to room temperature and a quantitative ^{1}H NMR experiment at 25 ºC was conducted on a Varian 500 MHz spectrometer (Xsen5mm probe with 1 H S/N of 1245.3) with the following parameters: number of scans (nt = 1), receiver gain = 30, and pulse width pw = 90. The equilibrium constants for each compound were measured using variable temperature NMR on a Bruker 600 MHz spectrometer. The sample was heated to each specified temperature for approximately 30 min followed by quantitative ^{1}H NMR analysis. The equilibrium constants were calculated from the relative integration of the ***S**$_a$*- to ***R**$_a$*-atropdiastereomer (see Fig below exemplified for ***R**$_a$***-1**/***S**$_a$***-1**).

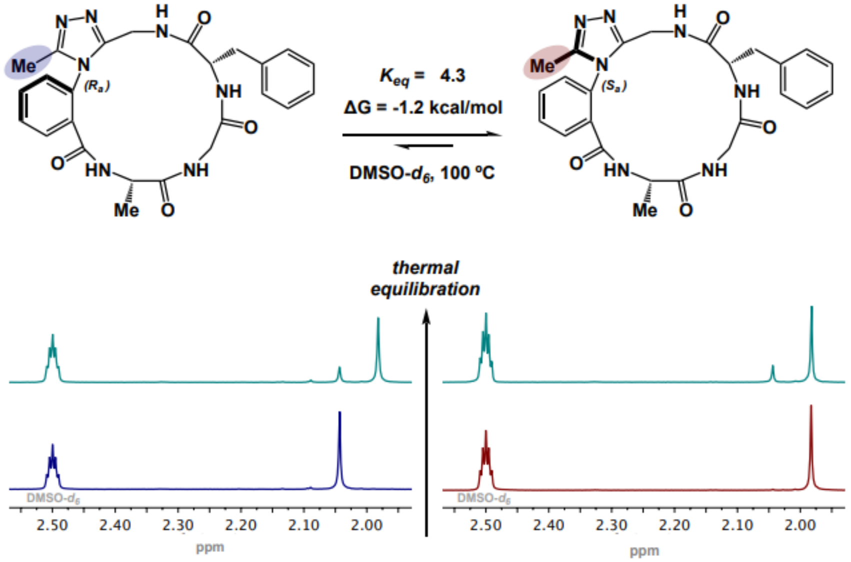

^{1}H NMR spectra (zoomed into Trz1-CH_3 group) of ***R*a-1** (blue) and ***S*a-1** (red) at room temperature. ^{1}H NMR spectrums (cyan) after heating to 100 ºC for 24 h. Equilibrium constant of ~4.3 measured for each system.

Macrocycle	Temperature (°C)	K_{eq}	ΔG (kcal/mol)
***R*a-1 → *S*a-1**	120±3.6	4.30 ± 0.172	-1.15 ± 0.09
***R*a-2 → *S*a-2**	120±3.6	2.02 ± 0.081	-0.55 ± 0.07

Summary of the equilibrium constants (K_{eq}) and standard Gibbs free energy differences for macrocycles determined at specific temperatures. Accuracy of temperature (± 3%) and ^{1}H NMR integration (± 4%) measurements were estimated and propagated through calculations. Uncertainty values in kcal/mol reported as ± 2*standard error.

Van't Hoff plot analysis: A graph of the natural logarithm of the equilibrium constants with respect to the inverse temperature (K^{-1}) was plotted and fitted to the linear form of the van't Hoff equation (eq. 1). The change in enthalpy and entropy for the atropisomerization process was estimated from the slope and intercept of the van't Hoff plot.

$$lnK_{eq} = -\frac{\Delta H}{RT} + \frac{\Delta S}{R} \quad \text{Eq. 1}$$

Analysis for R_a-1/S_a-1:

ΔH° (kJ/mol)	ΔS° (J/Kmol)
5.53	24.87

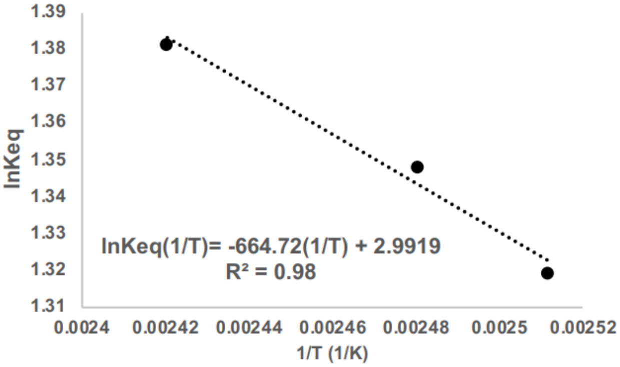

Temp. (K)	Keq [R/S]	1/T	lnKeq
398.15	3.74	0.00251162	1.31908561
403.15	3.85	0.00248047	1.34807315
413.15	3.98	0.00242043	1.38128182

Slope	-664.71827
y-intercept	2.99189068
Slope*-R	5526.46769
y-intercept*R	24.8745791
R-sqaured	0.98004407

Analysis for R_a-2/S_a-2:

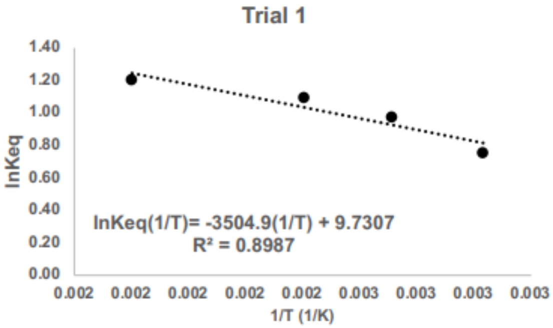

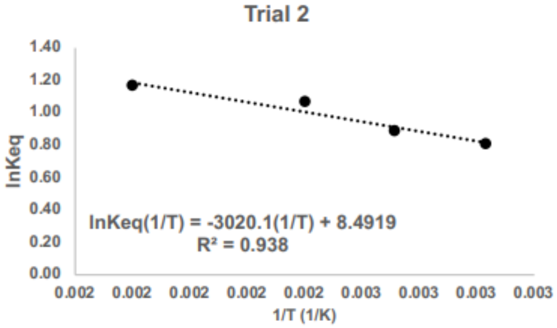

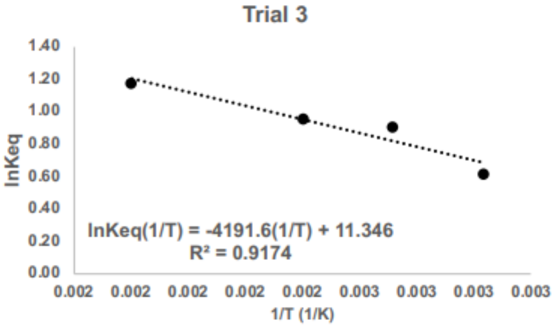

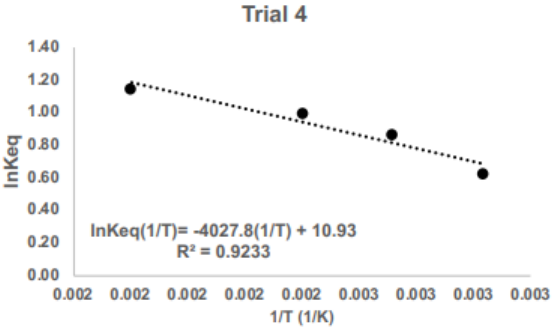

ΔH° (kJ/mol)	ΔS° (J/Kmol)
30.65 ± 4.42	84.18 ± 10.69

	Keq [S/R]			
Temp. (K)	Trial 1	Trial 2	Trial 3	Trial 4
393.15	2.12	2.22	1.85	1.86
398.15	2.66	2.42	2.47	2.38
403.15	2.98	2.89	2.61	2.69
413.15	3.34	3.18	3.23	3.13
	lnKeq			
1/T	Trial 1	Trial 2	Trial 3	Trial 4
0.00254356	0.75141609	0.79750720	0.61518564	0.62057649
0.00251162	0.97832612	0.88376754	0.90421815	0.86710049
0.00248047	1.09192330	1.06125650	0.95935022	0.98954119
0.00242043	1.20597081	1.15688120	1.17248214	1.14103300
Slope	-3504.9248	-3020.0915	-4191.6397	-4027.7641
y-intercept	9.73072783	8.49191334	11.3458729	10.9297375
Slope*-R	29139.9451	25109.041	34849.2923	33486.8307
y-intercept*R	80.9012712	70.6017675	94.3295875	90.8698376
R-sqaured	0.89871484	0.93800115	0.91740866	0.92326373

2 Appendix A: NMR Spectra

2.1 NMR Spectra of Dominant Rotors

2-(3-(((((9*H*-fluoren-9-yl)methoxy)carbonyl)amino)methyl)-5-methyl-4*H*-1,2,4-triazol-4-yl)benzoic acid (*a*)

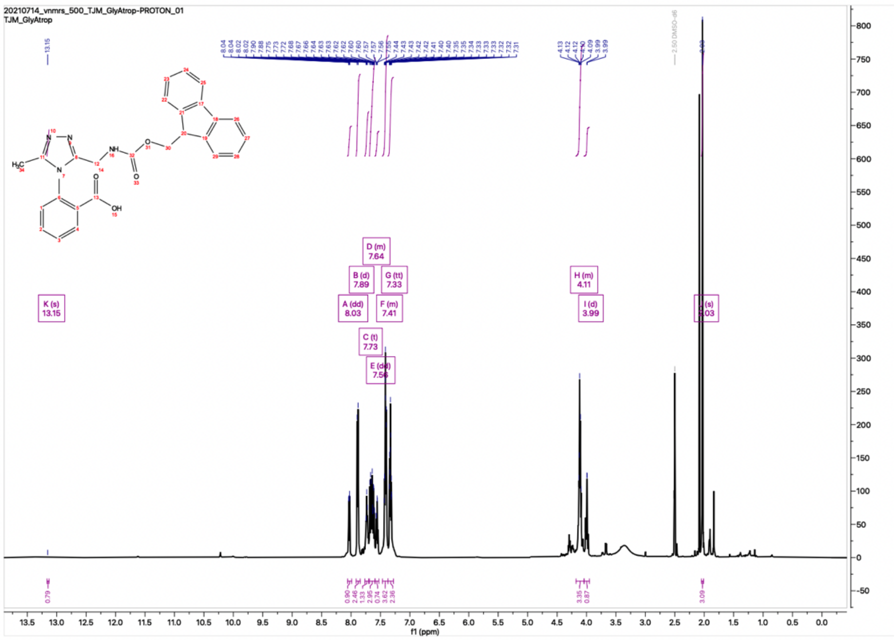

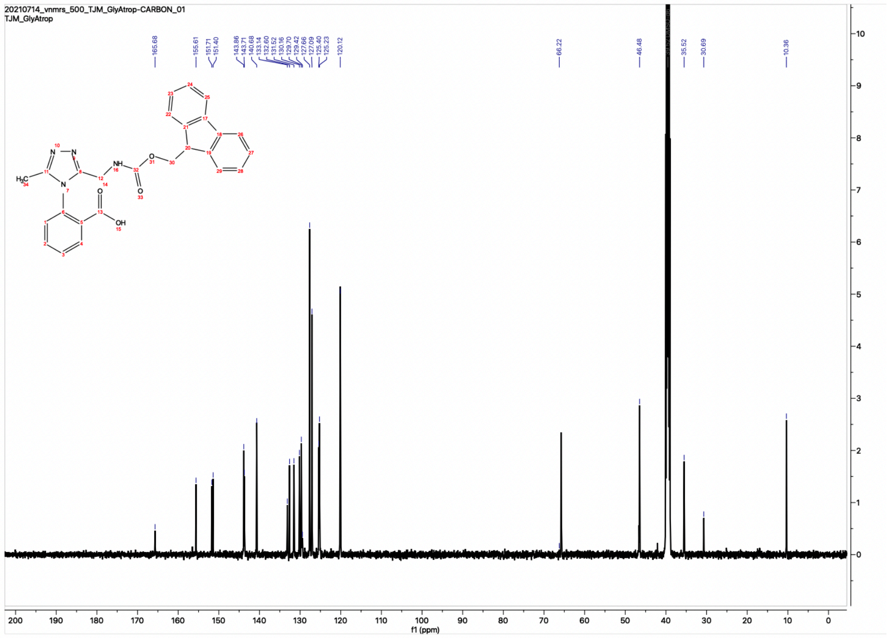

2-(3-(((((9*H*-fluoren-9-yl)methoxy)carbonyl)amino)methyl)-5-methyl-4*H*-1,2,4-triazol-4-yl)-6-methylbenzoic acid (*b*)

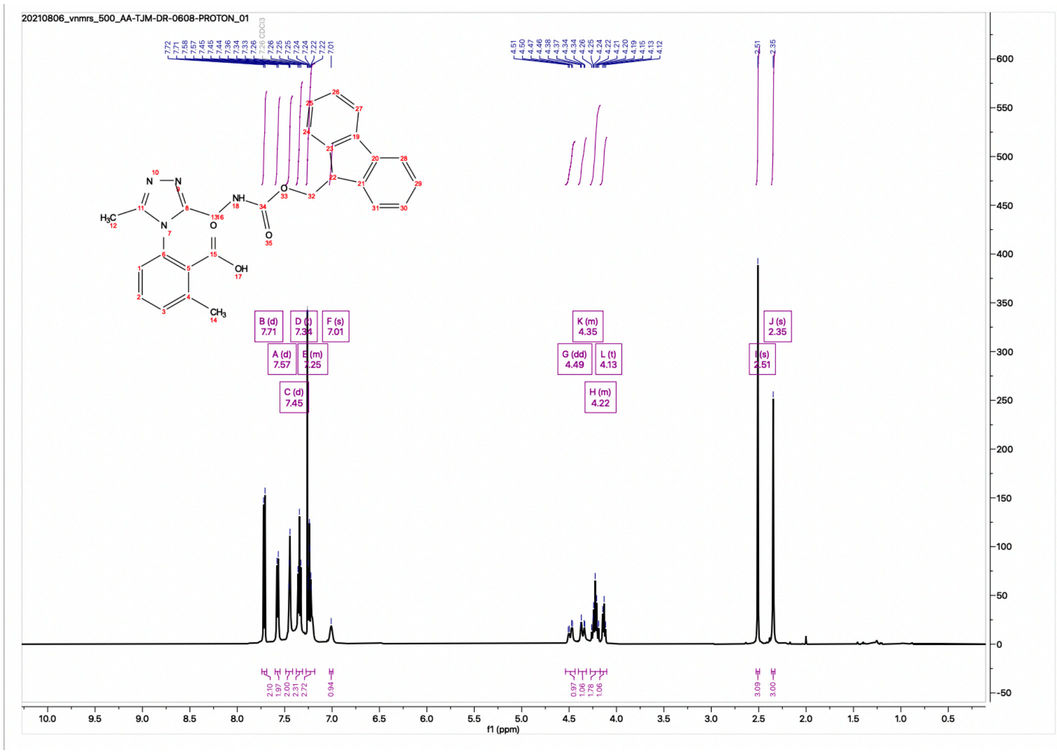

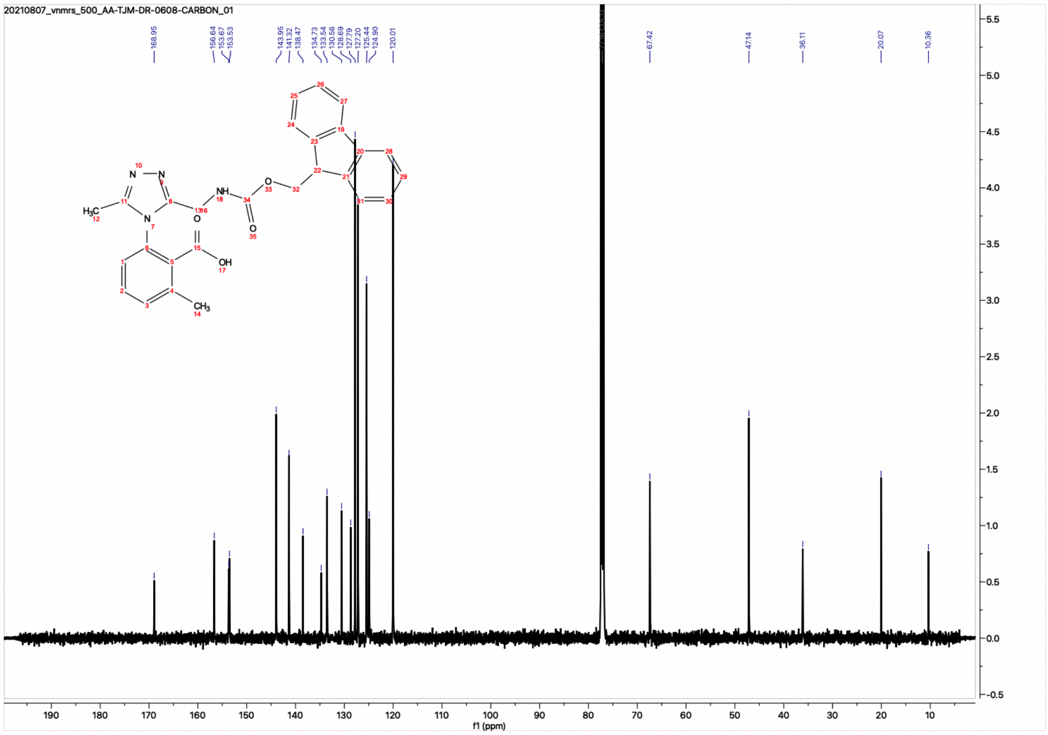

2-(3-((*R*)-1-((((9*H*-fluoren-9-yl)methoxy)carbonyl)amino)ethyl)-5-methyl-4*H*-1,2,4-triazol-4-yl)benzoic acid (*c*)

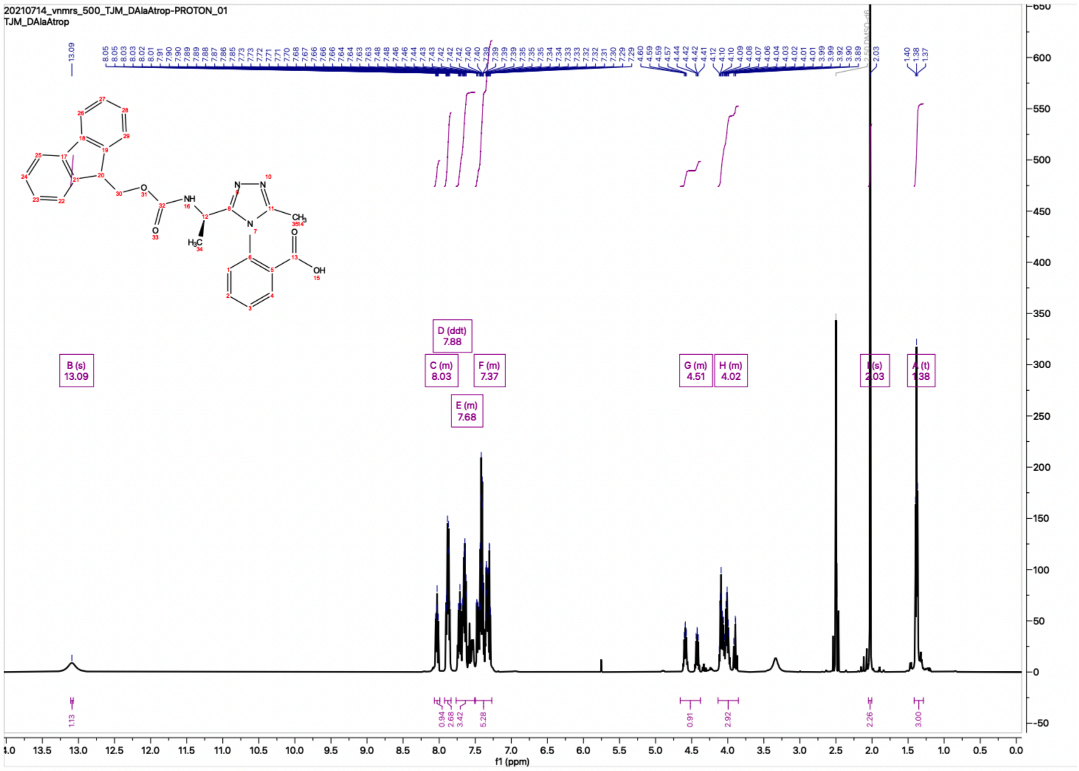

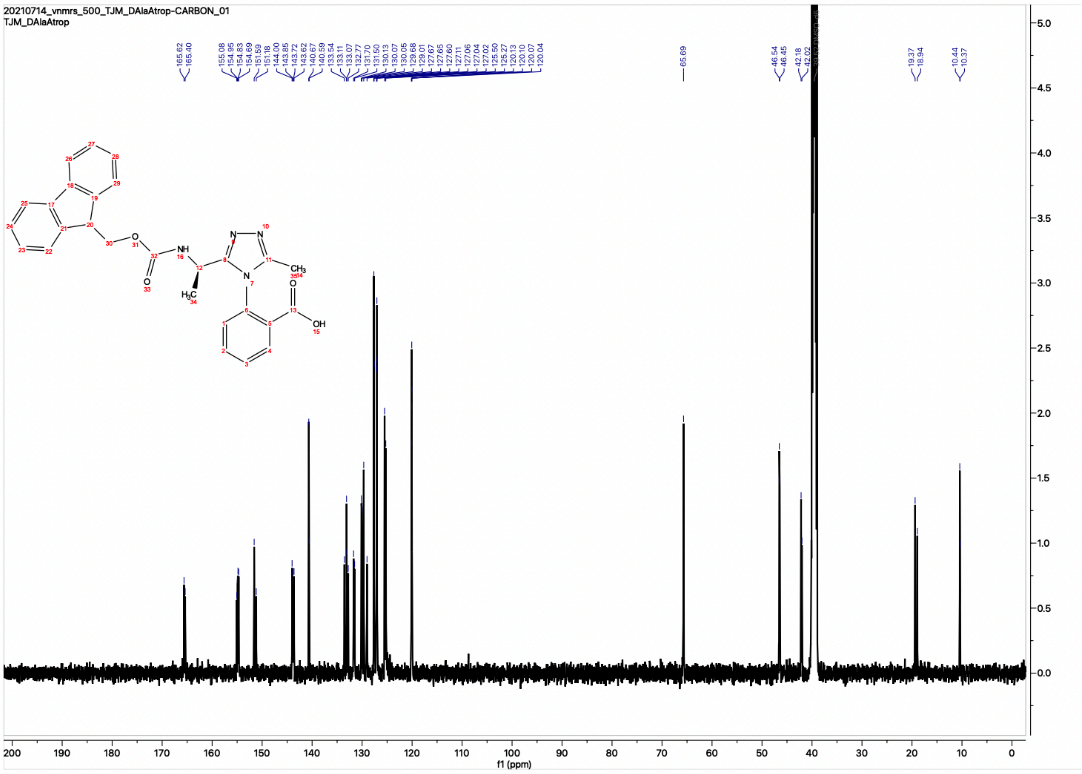

2-(3-((*S*)-1-((((9*H*-fluoren-9-yl)methoxy)carbonyl)amino)ethyl)-5-methyl-4*H*-1,2,4-triazol-4-yl)benzoic acid (*d*)

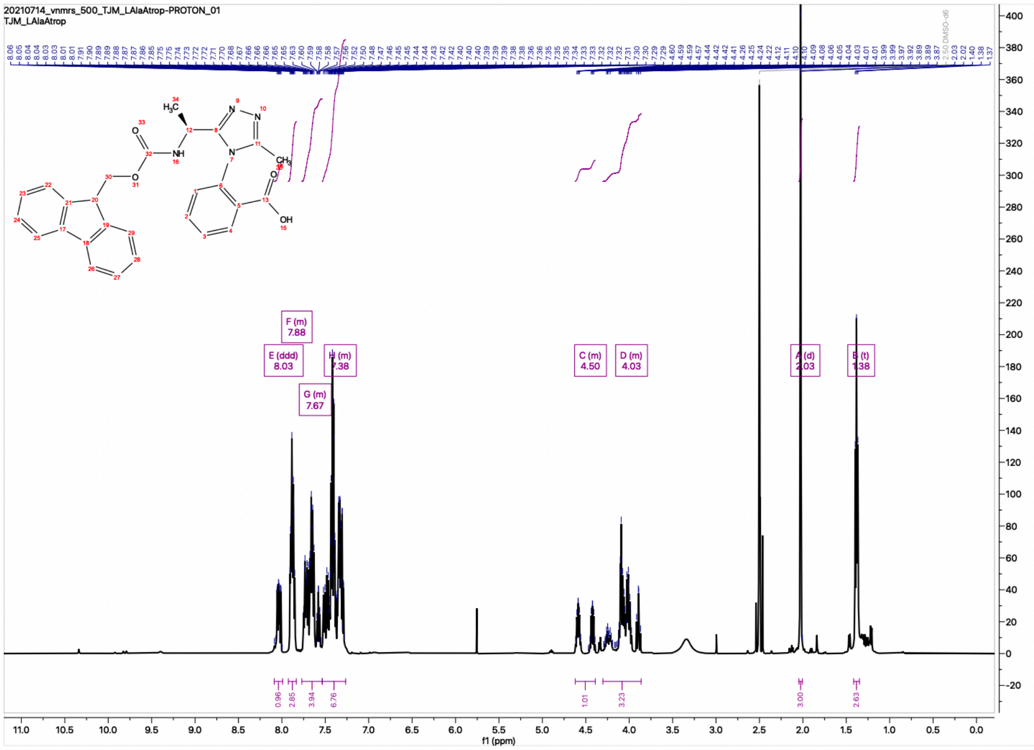

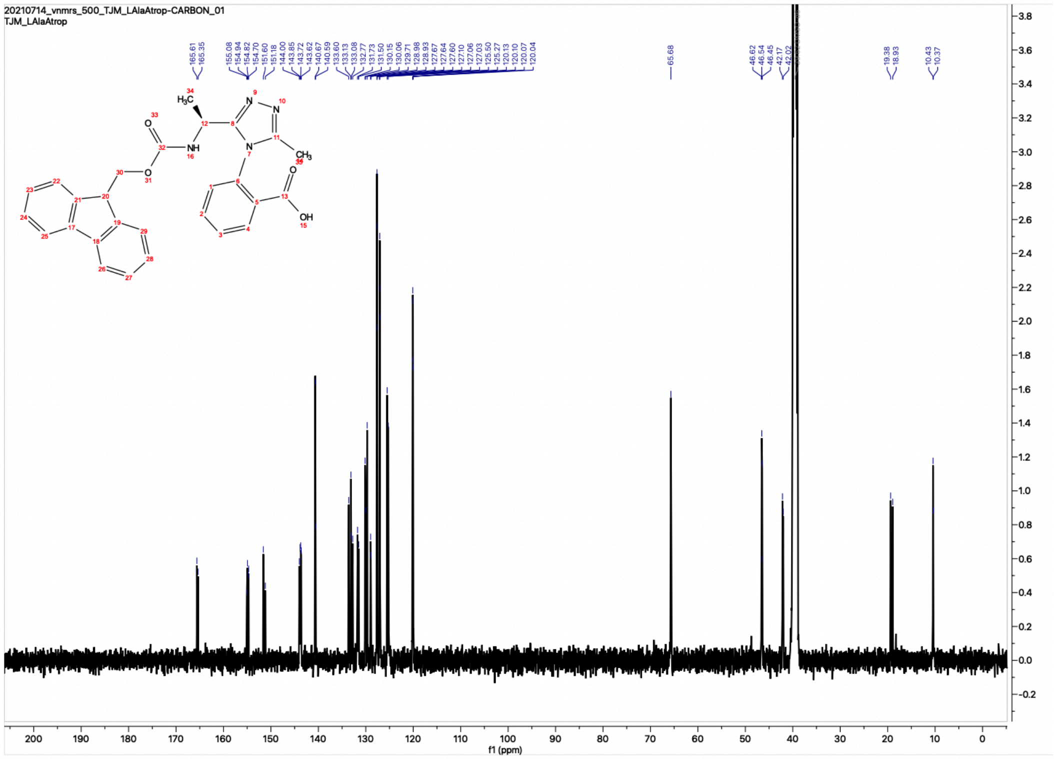

2.2 NMR Spectra of Macrocyclic Peptides

NMR spectra of ***R**$_a$***-1**, ***S**$_a$***-1**, ***R**$_a$***-2**, and ***S**$_a$***-2** are previously reported compounds and the experimental data can be found in the Supporting Information elsewhere.[2]

***cyclo*-[(*S*$_a$)-Trz(methyl/(*R*)-methyl)-Ala-Gly-Phe] (*S*$_a$-3)**

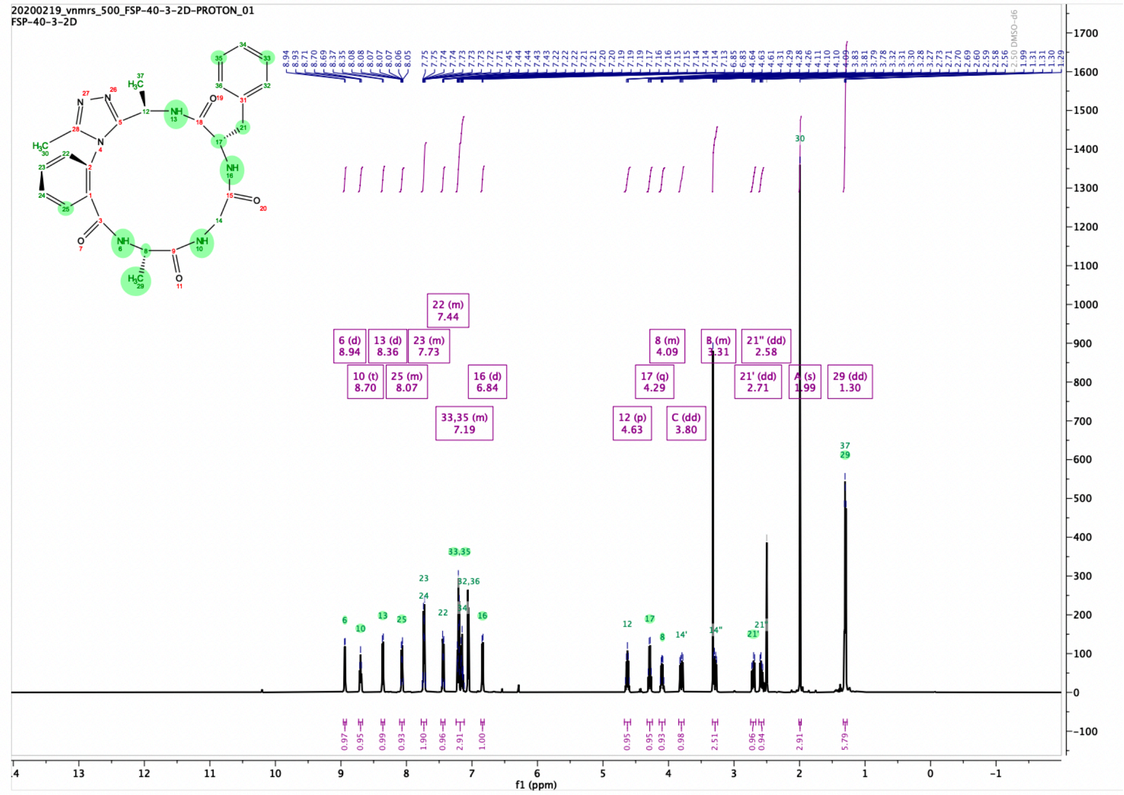

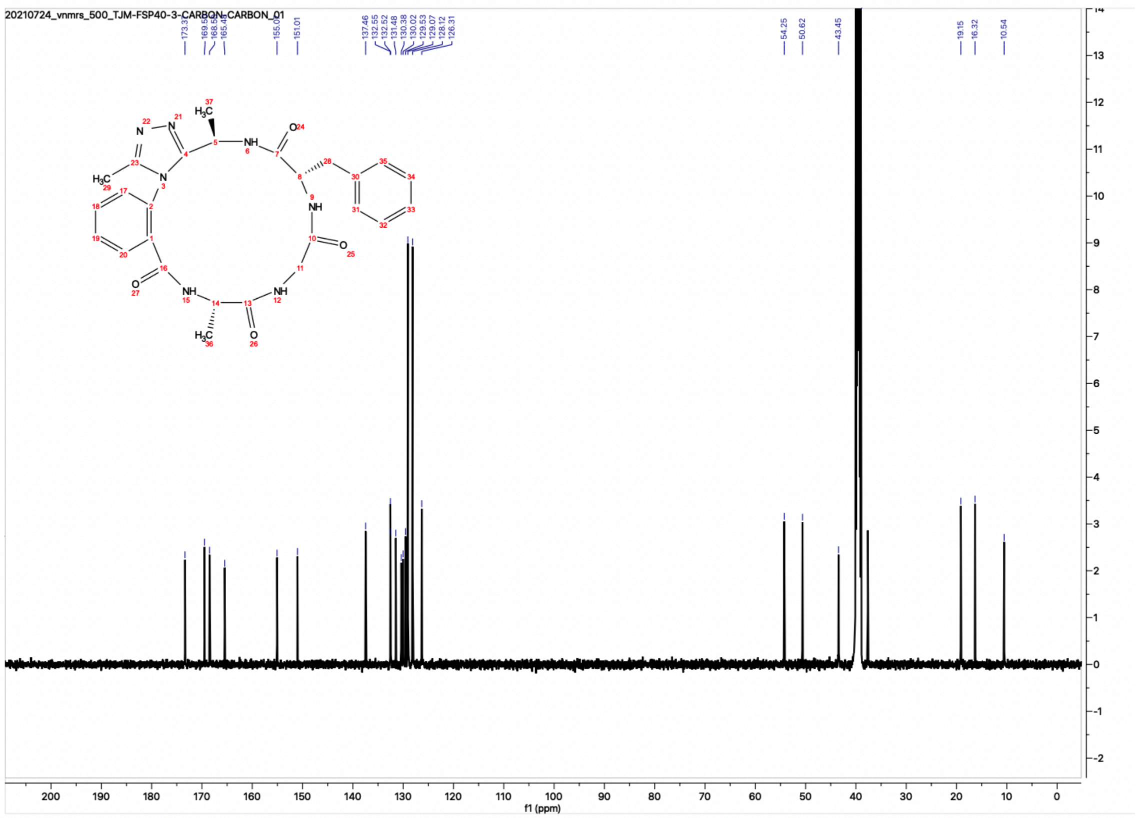

ROESY at 25 °C (S_a-3)

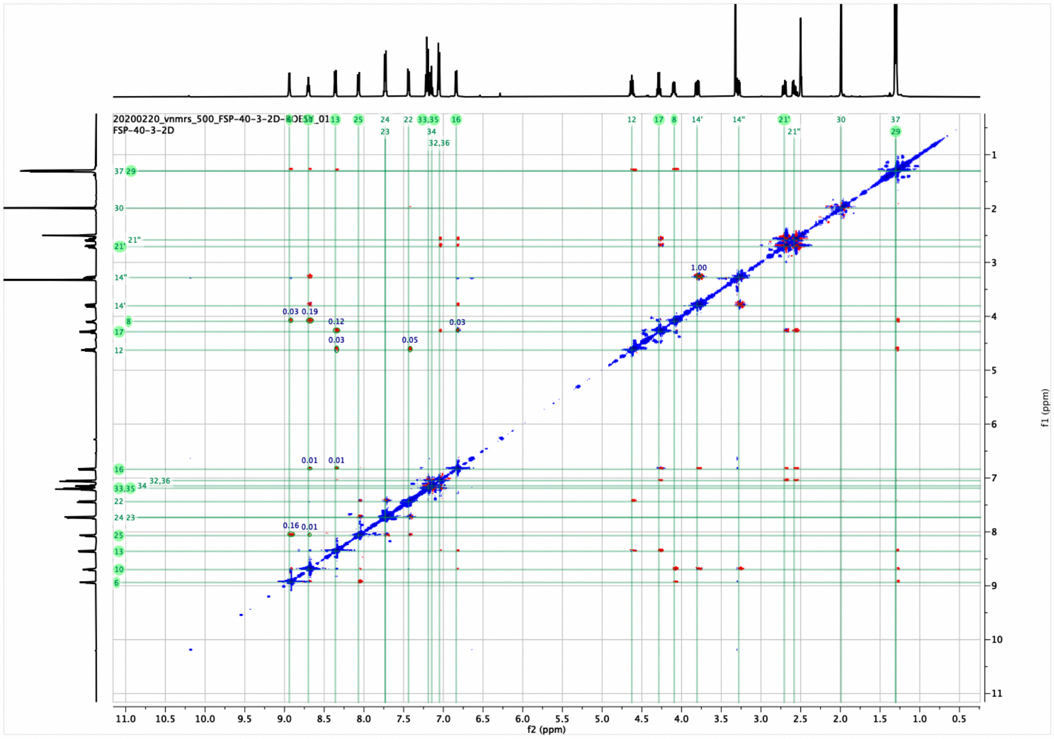

TOCSY at 25 °C (S_a-3)

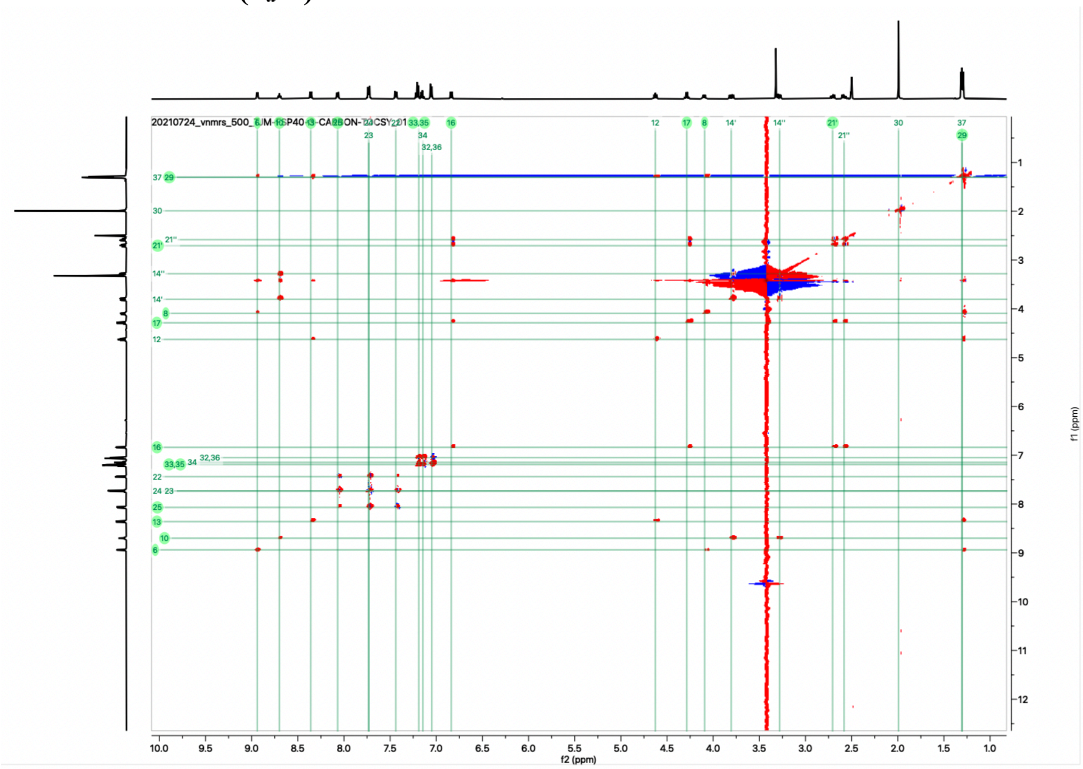

HSQC at 25 °C (S_a-3)

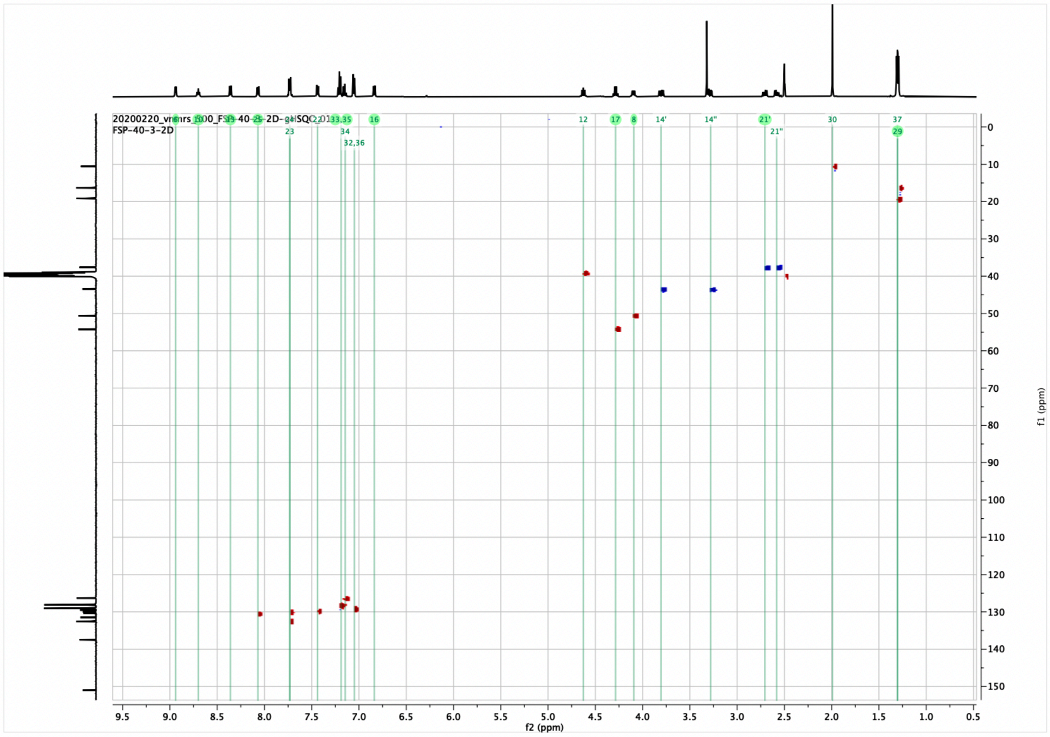

CIGARAD at 25 °C (S_a-3)

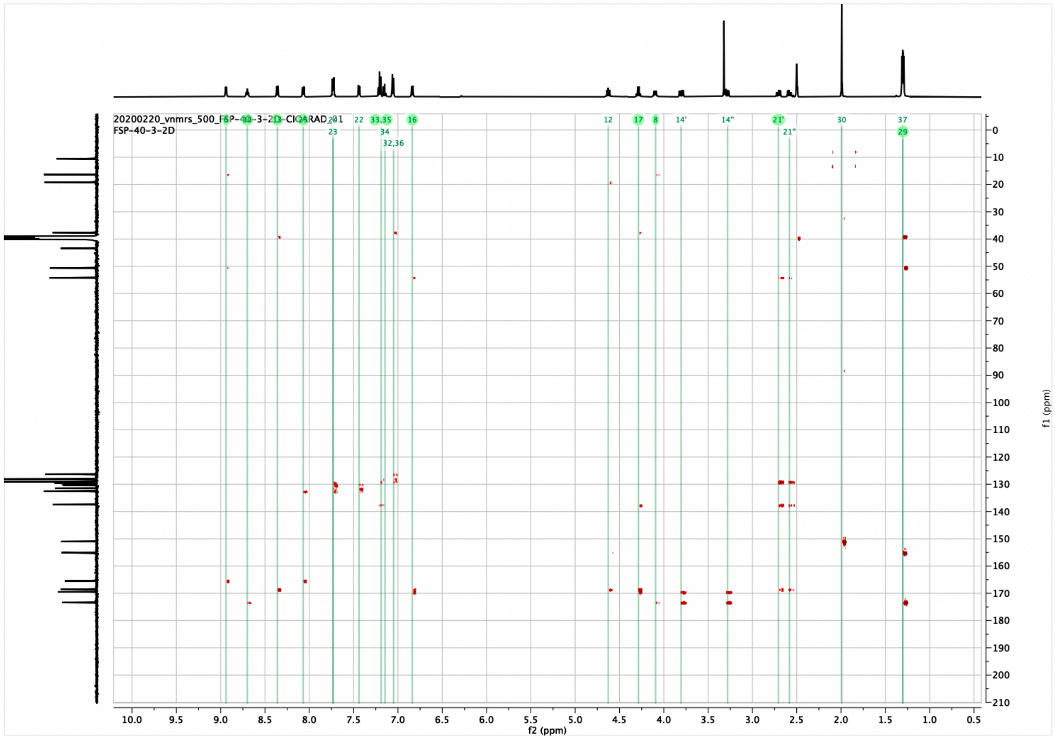

cyclo-[(R_a)-Trz(methyl/(S)-methyl)-Ala-Gly-Phe] (R_a-4)

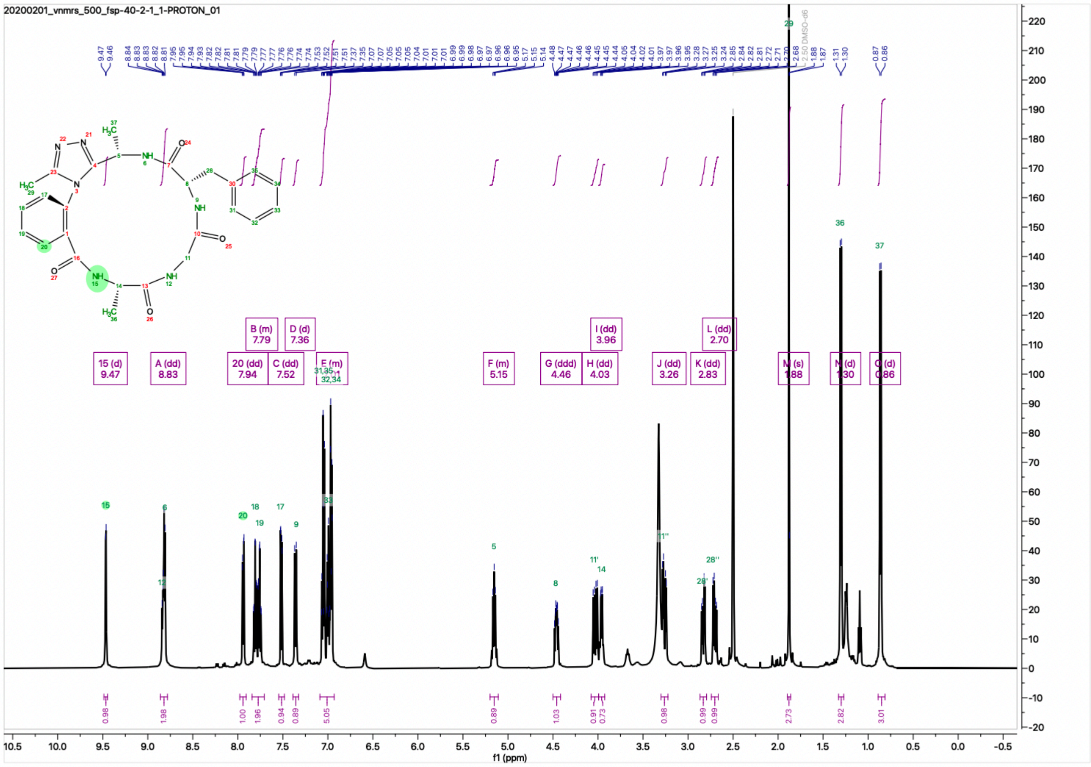

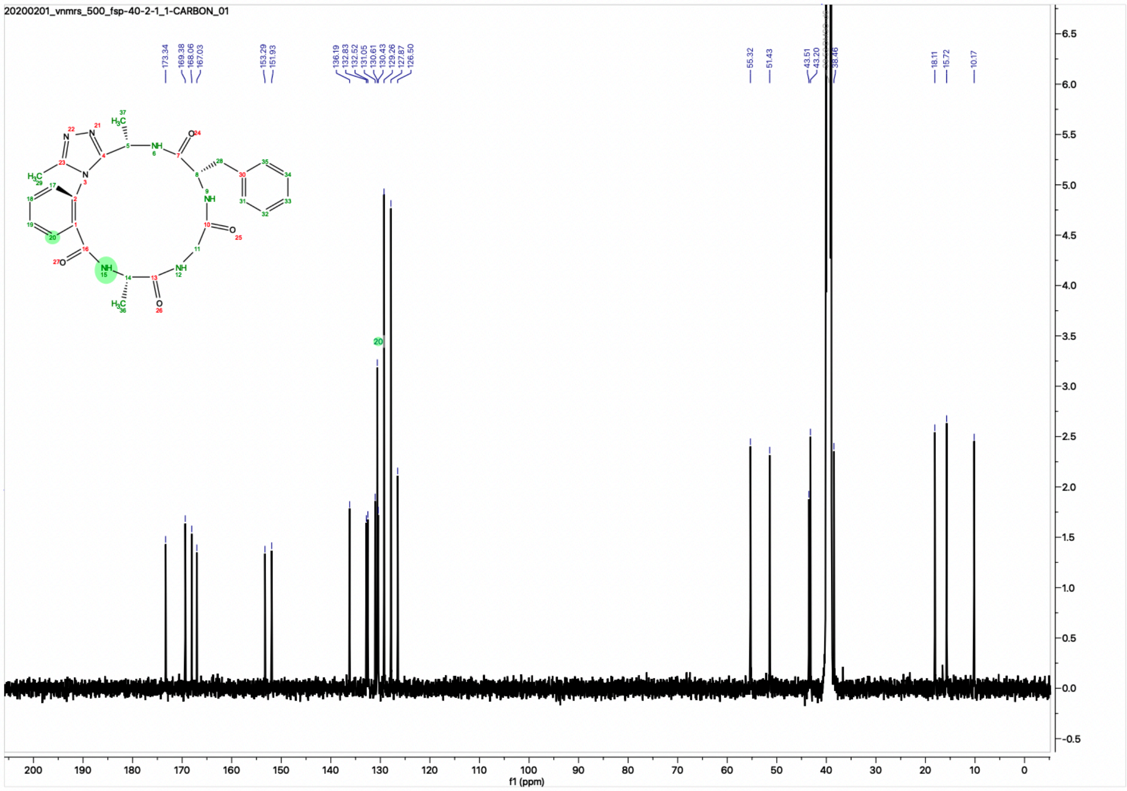

ROESY at 25 °C (R_a-4)

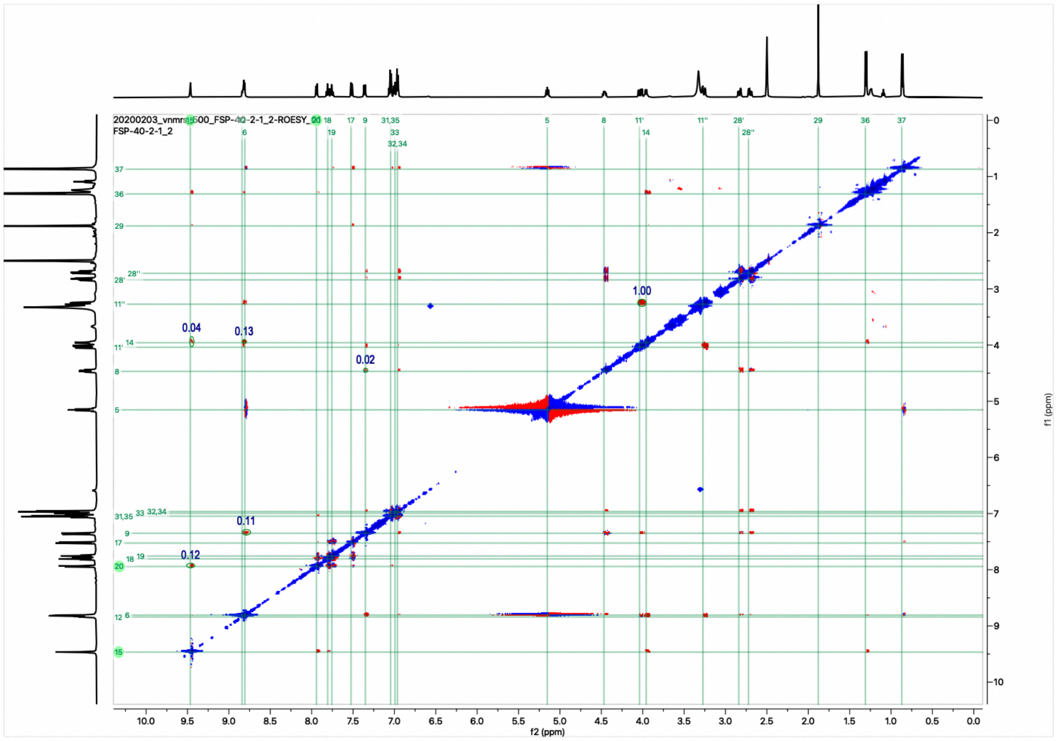

COSY at 25 °C (R_a-4)

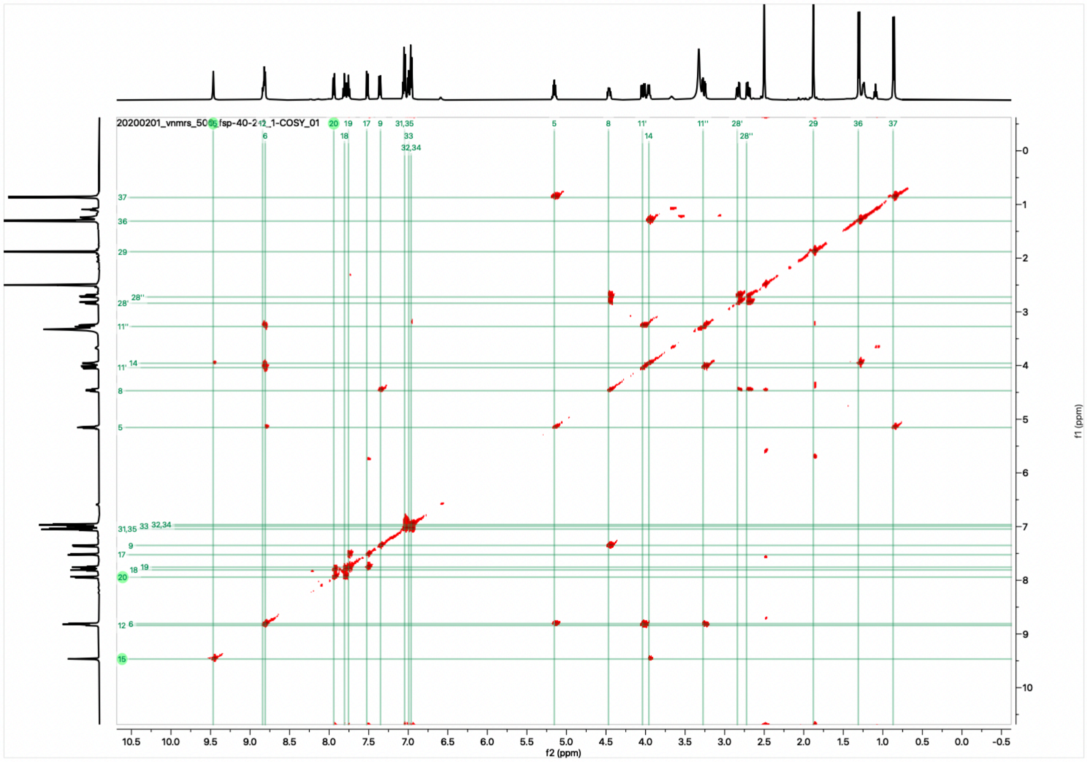

TOCSY at 25 °C (R_a-4)

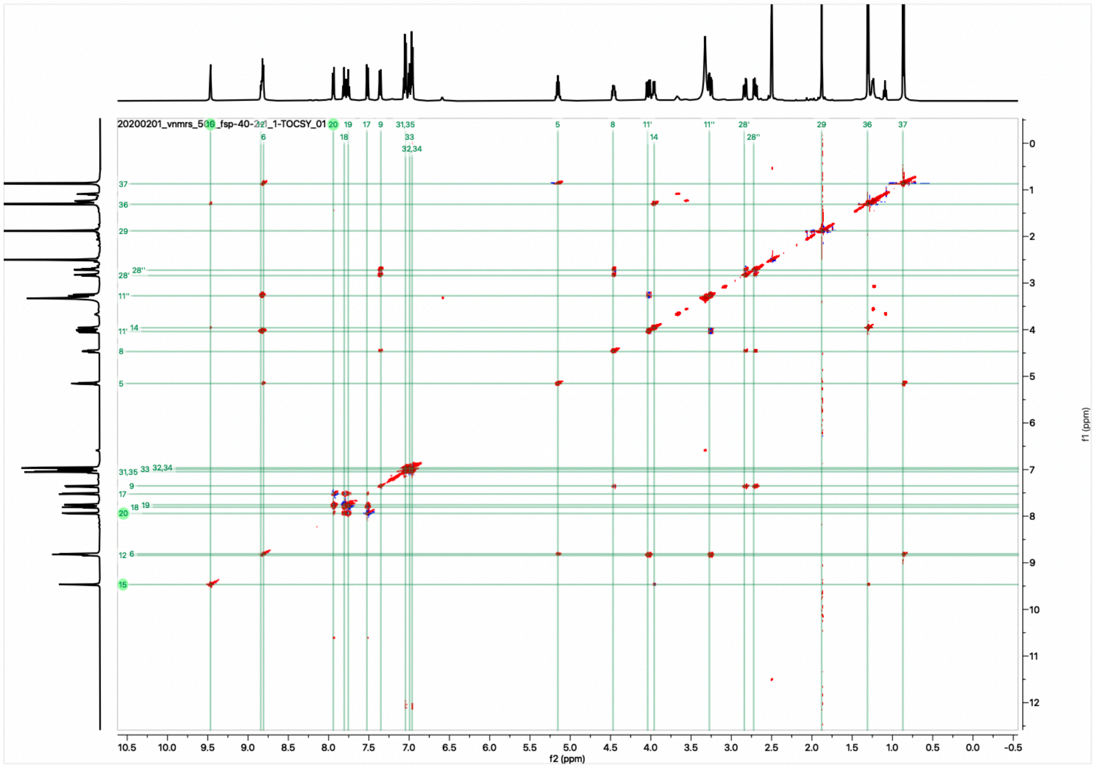

gHSQC at 25 °C (*R*$_a$-4)

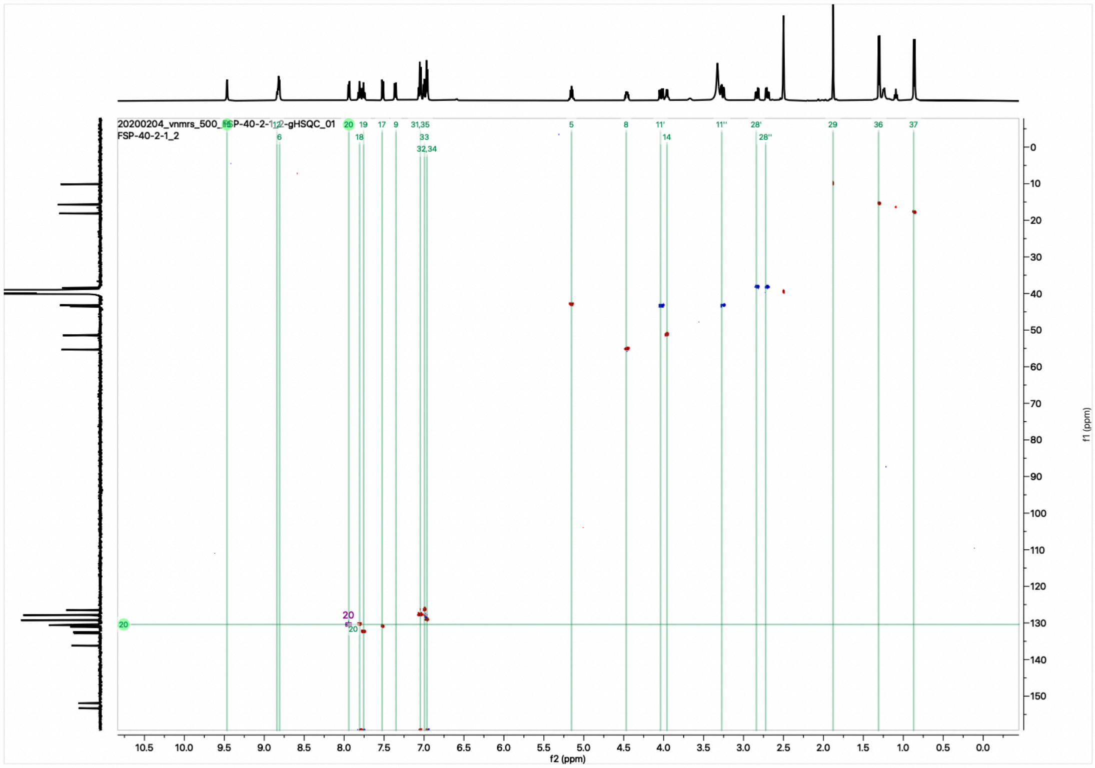

CIGARAD at 25 °C (*R*$_a$-4)

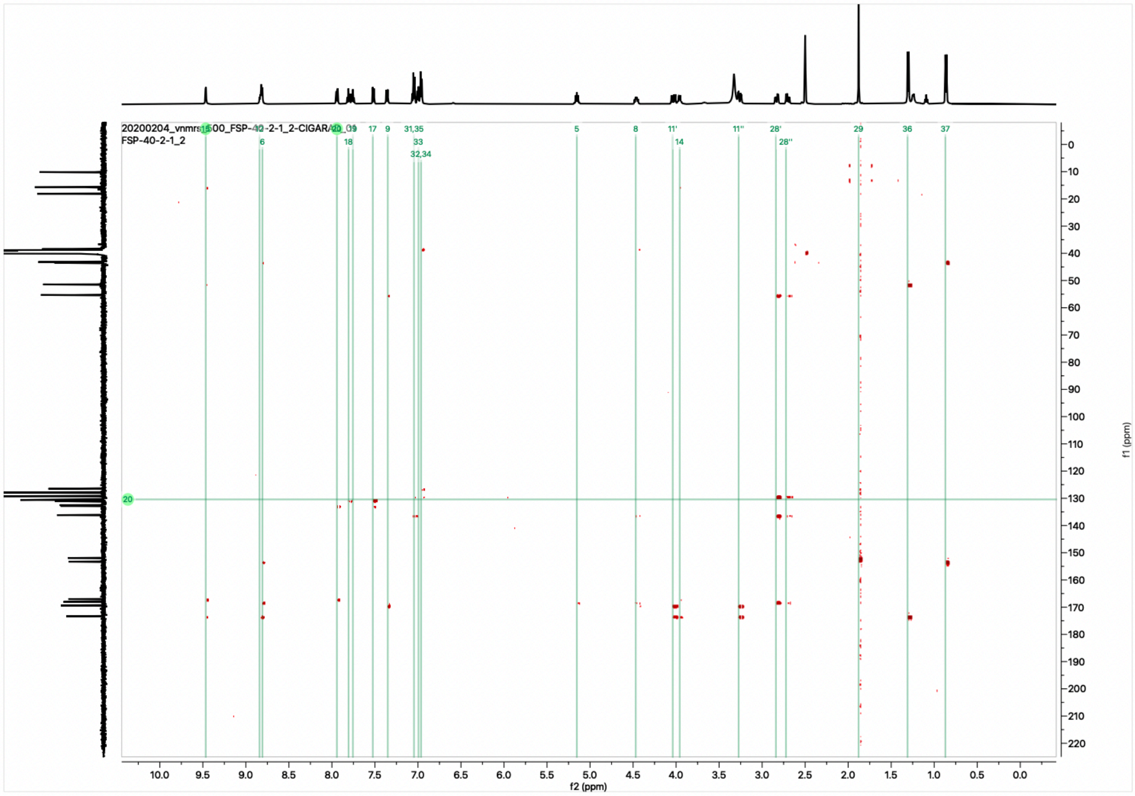

2.3 References

[1] Nagase, T.; Mizutani, T.; Ishikawa, S.; Sekino, E.; Sasaki, T.; Fujimura, T.; Ito, S.; Mitobe, Y.; Miyamoto, Y.; Yoshimoto, R.; Tanaka, T.; Ishihara, A.; Takenaga, N.; Tokita, S.; Fukami, T.; Sato, N. *J. Med. Chem.* **2008**, 51, 15, 4780-4789.

[2] Diaz, D. B.; Appavoo, S. D.; Bogdanchikova, A. F.; Lebedev, Y.; McTiernan, T. J.; dos Passos Gomes, G.; Yudin, A. K. *Nat. Chem.* **2021**, *13*, 218-225.

[3] Appavoo, S. D.; Kaji, T.; Frost, J. R.; Scully, C. C. G.; Yudin, A. K. *J. Am. Chem. Soc.* **2018**, 140, 28, 8763–8770.
